# The B cell transcription program mediates hypomethylation and overexpression of key genes in Epstein-Barr virus-associated proliferative conversion

**DOI:** 10.1186/gb-2013-14-1-r3

**Published:** 2013-01-15

**Authors:** Henar Hernando, Claire Shannon-Lowe, Abul B Islam, Fatima Al-Shahrour, Javier Rodríguez-Ubreva, Virginia C Rodríguez-Cortez, Biola M Javierre, Cristina Mangas, Agustín F Fernández, Maribel Parra, Henri-Jacques Delecluse, Manel Esteller, Eduardo López-Granados, Mario F Fraga, Nuria López-Bigas, Esteban Ballestar

**Affiliations:** 1 Chromatin and Disease Group, Cancer Epigenetics and Biology Programme (PEBC), Bellvitge Biomedical Research Institute (IDIBELL), Avda. Gran Via 199-203, 08908 L'Hospitalet de Llobregat, Barcelona, Spain; 2 CR-UK Institute for Cancer Studies, University of Birmingham, Vincent Drive, Birmingham B15 2TT, UK; 3 Department of Experimental and Health Sciences, Barcelona Biomedical Research Park, Universitat Pompeu Fabra (UPF), c/Dr. Aiguader, 88, 08003 Barcelona, Spain; 4 Broad Institute, 7 Cambridge Center, Cambridge, MA 02142, USA and Hematology Division, Brigham and Women's Hospital, Harvard Medical School, One Blackfan Circle, Brookline, MA 02115, USA; 5 Cancer Epigenetics Laboratory, Instituto Universitario de Oncología del Principado de Asturias (IUOPA), HUCA, Universidad de Oviedo, C/Dr. Emilio Rodríguez Vigil, s/n, 33006 Oviedo, Spain; 6 Cellular Differentiation Group, Cancer Epigenetics and Biology Programme (PEBC), Bellvitge Biomedical Research Institute (IDIBELL), Avda. Gran Via 199-203, 08908 L'Hospitalet de Llobregat, Barcelona, Spain; 7 Department of Virus Associated Tumours, German Cancer Research Center, Im Neuenheimer Feld 280, 69120 Heidelberg, Germany; 8 Cancer Epigenetics Group, Cancer Epigenetics and Biology Programme (PEBC), Bellvitge Biomedical Research Institute (IDIBELL), Avda. Gran Via 199-203, 08908 L'Hospitalet de Llobregat, Barcelona, Spain; 9 Clinical Immunology Department, University Hospital La Paz, P° de la Castellana, 261, 28046 Madrid, Spain; 10 Department of Immunology and Oncology, Centro Nacional de Biotecnología/CNB-CSIC, Cantoblanco, Darwin 3, 28049 Madrid, Spain

## Abstract

**Background:**

Epstein-Barr virus (EBV) infection is a well characterized etiopathogenic factor for a variety of immune-related conditions, including lymphomas, lymphoproliferative disorders and autoimmune diseases. EBV-mediated transformation of resting B cells to proliferating lymphoblastoid cells occurs in early stages of infection and is an excellent model for investigating the mechanisms associated with acquisition of unlimited growth.

**Results:**

We investigated the effects of experimental EBV infection of B cells on DNA methylation profiles by using high-throughput analysis. Remarkably, we observed hypomethylation of around 250 genes, but no hypermethylation. Hypomethylation did not occur at repetitive sequences, consistent with the absence of genomic instability in lymphoproliferative cells. Changes in methylation only occurred after cell divisions started, without the participation of the active demethylation machinery, and were concomitant with acquisition by B cells of the ability to proliferate. Gene Ontology analysis, expression profiling, and high-throughput analysis of the presence of transcription factor binding motifs and occupancy revealed that most genes undergoing hypomethylation are active and display the presence of NF-κB p65 and other B cell-specific transcription factors. Promoter hypomethylation was associated with upregulation of genes relevant for the phenotype of proliferating lymphoblasts. Interestingly, pharmacologically induced demethylation increased the efficiency of transformation of resting B cells to lymphoblastoid cells, consistent with productive cooperation between hypomethylation and lymphocyte proliferation.

**Conclusions:**

Our data provide novel clues on the role of the B cell transcription program leading to DNA methylation changes, which we find to be key to the EBV-associated conversion of resting B cells to proliferating lymphoblasts.

## Background

Infection of B cells with Epstein-Barr virus (EBV), which is highly prevalent in humans, is an excellent model to investigate the molecular mechanisms associated with the acquisition of unlimited growth during disease. EBV-associated changes in B cells are relevant to the development and progression of lymphomas [1] and lymphoproliferative disorders in immune-suppressed individuals, and various autoimmune disorders like rheumatoid arthritis, systemic lupus erythematosus and multiple sclerosis [2]. In early primary human infection, EBV infects peripheral resting B cells and expresses six nuclear (EBNA1, 2, 3A, 3B, 3C and -LP) and two latent membrane proteins and small non-coding RNAs. This type of infection, in which these two groups of proteins transform resting B lymphocytes into continuously proliferating lymphoblastoid cell lines, is referred to as type III latency [1,3]. This process mimics antigen-induced clonal expansion of resting B cells associated with MYC-mediated proliferation and upregulation of NF-κB, MAP kinases and antiapoptotic factors. Recent data have shown that EBNA2, which is essential to this process, enhances and exploits the B cell transcription program by binding to a variety of B cell transcription factor sites to achieve transformation [4]. *In vivo*, the vigorous cellular immune response directed against EBV-immortalized cells limits the proliferation and expansion of such latently infected cells at early stages of infection of a naïve host or in immunocompromised individuals. Studying type III latency lymphoblastoid cells is relevant because it not only allows the investigation of early steps in infection and the effects that the viral activity exerts on B cell function, but also is an excellent strategy for investigating changes related to the triggering of unlimited proliferation of B cells, before any additional secondary transforming genetic and epigenetic events occur.

The mechanisms by which B cell identity is altered in this process towards unlimited proliferation, triggered by EBV infection, involve the acquisition of epigenetic changes. In this context, DNA methylation might play a key role, since this epigenetic mark participates in regulating transcriptional activity [5] and is known to be highly aberrant in several types of EBV-associated lymphomas [6,7] and autoimmune diseases [8]. Despite its role in gene control, DNA methylation is not only a mechanism of transcriptional control but also guarantees genomic stability. The relationship between methylation and transcriptional activity has been best studied in promoter regions, particularly CpG island-associated promoters, where methylation is generally associated with transcriptional repression. In the context of the hematopoietic system, DNA methylation profiling has revealed overall higher methylation levels in the lymphoid branch relative to the myeloid one, and with respect to less differentiated progenitors [9].

A number of studies have addressed the analysis of DNA methylation changes associated with EBV infection of B cells. Several of these have revealed that whereas the EBV genomic sequence is virtually unmethylated in free viral particles and lymphoblastoid cells, the genome is heavily methylated in both Burkitt and Hodgkin lymphomas [10]. Also, the DNA methylation status of EBV promoters has been widely studied in association with the activity of latency promoters [11-14]. By contrast, fewer studies have addressed the acquisition of DNA methylation changes by the host cell during EBV-mediated transformation between resting B cells and proliferating lymphoblasts. EBV influences changes in the DNA methylation status at specific sequences [15-17] and these are likely to influence or modify the B cell phenotype and function. It is therefore of inherent interest to investigate the extent and mechanisms of acquisition of changes in DNA methylation by B cells following EBV infection as well as their potential contribution to phenotypic changes during this process.

In this study we investigated the acquisition of DNA methylation changes during EBV-mediated transformation of resting B cells to lymphoblastoid cell lines by using methylation bead arrays. We exclusively observed significant hypomethylation of around 250 genes. No hypermethylation was found. Time course analysis indicated that hypomethylation occurs only when cell proliferation has started, suggesting the exclusive participation of replication-dependent mechanisms. Gene Ontology (GO) analysis, comparison with the expression patterns of different cell types and between resting B cells and proliferating lymphoblasts, high-throughput analysis of the presence of transcription factor binding motifs and occupancy revealed that most genes undergoing hypomethylation are active and display the presence of NF-κB p65 and other B cell-specific transcription factors. In addition, hypomethylation associates with upregulation of several genes that are essential in the transformation of resting B cells to continuously proliferating lymphoblasts. Pharmacologically induced DNA demethylation increases B cell transformation efficiency. Our data provide novel clues to the contribution of epigenetic mechanisms associated with EBV-associated conversion of resting B cells to proliferating lymphoblasts, the relevance of the cell type context and why DNA hypomethylation could be key in the efficiency of this process.

## Results

### DNA methylation profiling reveals that EBV-mediated B cell to lymphoblastoid transformation is associated with gene-specific hypomethylation

**Figure 1:**
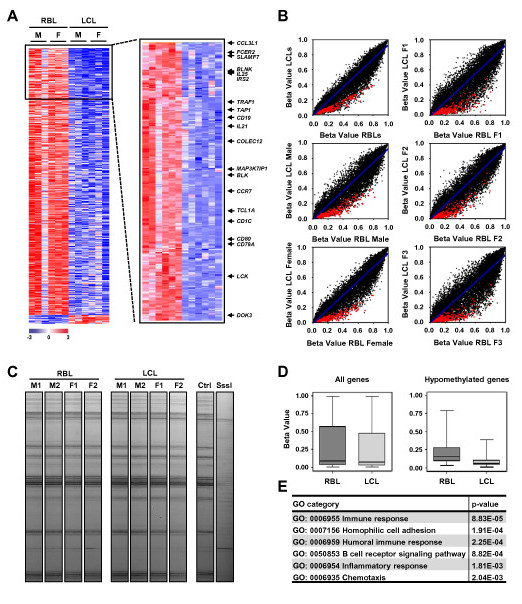
**High-throughput methylation comparison of resting B lymphocytes and matching lymphoblastoid cells**. **(a) **Heatmaps including the data for the six RBL/LCL pairs of samples showing significant differential methylation. The left panel shows all the genes showing a FDR < 0.05. Only those genes with a FC > 2 were selected (right panel). Data from the Illumina array were normalized as xi = xi - row.mean[i])/(0.333 × row.sd[i]). A scale is shown at the bottom, whereby positive (red) and negative (blue) values correspond, respectively, to higher and lower than average methylation status. M, male; F, female. **(b) **Scatterplots showing methylation profiles of matching RBL/LCL pairs. Genes with significant differences (FC > 2, FDR < 0.05) in averaged results from six samples are highlighted in red. Six panels are shown: top left, mean of six experiments/pairs of samples; middle left, male samples; bottom left, female samples; right panels, three individual RBL/LCL comparisons. **(c) **Band patterning corresponding to the analysis of unmethylated/methylated Alu (AUMA) repeats. Four RBL/LCL (M, male; F, female) pairs are shown. To illustrate the sensitivity toward DNA methylation changes, a B cell sample is compared with the same sample following limited treatment with SssI DNA methyltransferase. **(d) **Comparison of the methylation levels in RBLs and LCLs for all the CpGs represented in the 27k bead array and CpGs that undergo hypomethylation in this process. Box and whisker plots are presented, where the bottom and top of the box are the 25th and 75th percentile and the bar near the middle is the 50th percentile (the median). **(e) **Gene Ontology (GO) analysis of hypomethylated genes during EBV-mediated RBL to LCL conversion.

To investigate the acquisition of DNA methylation changes in association with EBV-associated transformation of resting B lymphocytes (RBLs), we first compared the DNA methylation profiles of six samples before and after EBV infection, once they had become lymphoblastoid cell lines (LCLs). To this end, we used methylation bead arrays that interrogate the DNA methylation status of over 27,000 informative CpG sites, including the region near the transcription start sites of more than 14,000 promoters. Statistical analysis of the combined data from the six pairs of samples revealed that 256 genes were hypomethylated (fold change (FC) > 2; false discovery rate (FDR) adjusted *P *< 0.05, Student's *t*-test) in B lymphoblastoid cells compared with resting B cells (Figure 1a; Additional file 1). By contrast, no hypermethylated genes were observed under these conditions. Scatterplots comparing the average DNA methylation patterns of the six RBLs and matching LCLs had highly reproducible DNA methylation profiles among different samples (Figure 1b). Changes corresponding to the average six pairs of B cells/lymphoblastoid cells were almost identical to the pattern obtained for only male or only female RBL/LCL comparisons, or those changes obtained for each individual pair of samples (Figure 1b), highlighting the specificity of the differences observed.

Since disease-related hypomethylation alterations generally affect repetitive elements, we also investigated changes in CpG sites in this type of sequence. In humans, most of the methylated cytosines are found in CpG-rich sequences within the tandem and interspersed repeats that constitute up to 45% of the human genome, of which Alu repeats are the most common family. We used genome-wide amplification of unmethylated DNA Alu repeats (AUMA) [18] to perform high-throughput screening of DNA methylation at these sequences. This experiment revealed no differences between RBLs and LCLs, suggesting that loss of methylation does not occur in repetitive sequences (Figure 1c).

Our analysis revealed a mean 21.5% methylation among the 256 genes undergoing demethylation in RBLs (Figure 1d). This average level of methylation at or near promoters is compatible with active gene expression in resting B cells. In fact, GO analysis showed significant enrichment (*P *< 0.05) for the following categories assigned to biological processes: immune response (GO:0006955; *P*-value = 8.8 × 10^-5^), humoral immune response (GO:0006959; *P*-value = 2.2 × 10^-4^), B cell receptor signaling pathway (GO:0050853; *P*-value = 8.8 × 10^-4^), inflammatory response (GO:0006954; *P*-value = 1.8 × 10^-3^) and chemotaxis (GO:0006935; *P*-value = 2.0 × 10^-3^) (Figure 1e). These categories include the presence of key markers of B cell function and identity, such as CD19, CD79a and BLNK. Among the list of hypomethylated genes, the presence of genes that are EBV-induced, such as *CCR7 *(EBI1), *GPR183 *(EBI2), *EBI3 *(IL27 subunit beta) and *TRAF1 *(EBI6), is also noteworthy.

**Figure 2:**
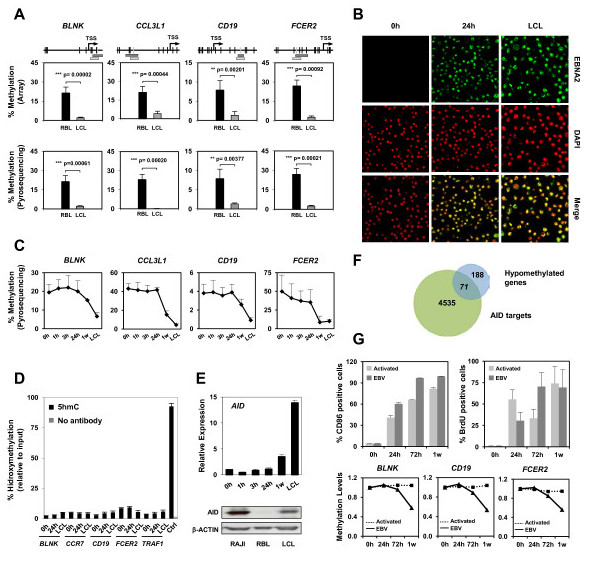
**A comparison of the DNA methylation levels of selected genes between RBLs and resulting LCLs**. **(a) **Bisulfite pyrosequencing was performed for the genes selected from experiments with methylation arrays. The distribution of CpG sites (vertical black line), the transcription start site (TSS; arrow) and the exact CpGs analyzed by the array (horizontal light grey bar) and pyrosequencing (horizontal dark grey bar) are indicated above each gene. Average data from six samples is represented. **(b) **EBNA2 and DAPI staining of B lymphocytes at 0 and 24 hours after infection with EBV derived from 293 cells carrying a recombinant B95.8 EBV genome, and LCLs, in which high levels of infection were achieved. **(c) **Pyrosequencing data showing the methylation changes at selected genes at different times. **(d) **Analysis of the 5-hydroxymethylcytosine content/changes at CpG sites undergoing hypomethylation in selected genes. As a positive control, *in vitro *hydroxymethylated DNA was used. **(e) **Changes in expression of activation-induced deaminase (AID) during RBL conversion to LCLs at the mRNA and protein levels. For protein analysis, the Burkitt's lymphoma cell line RAJI was used as a positive control for AID expression. **(f) **Overlap between AID target genes and hypomethylated genes. **(g) **Comparison between B cells activated with IL4 and CD40L and infected with EBV. The top panel shows the proportion of activated cells (left; determined by fluorescence-activated cell sorting (FACS) analysis with CD86) and proportion of dividing cells (right; determined with bromodeoxyuridine (BrdU)). Bottom, bisulfite pyrosequencing analysis of selected CpGs in association with EBV infection and with B cell activation by IL4 and CD40L. W, week.

To confirm that differences identified in DNA methylation between B cells and B lymphoblasts were robust, we carried out bisulfite genomic pyrosequencing of selected genes looking at CpG sites corresponding to the oligonucleotide probe represented in the methylation array, which is generally located around the transcription start site. We selected 20 genes on the basis of the magnitude of change in methylation, as revealed by the analysis of our methylation arrays, and their functional relevance in the context of B cell biology: *CD19*, *CD79a*, *BLK*, *FCER2*, *LCK*, *BLNK*, *IL21*, *CCL3L1*, *SLAMF7*, *IL25*, *IRS2*, *TAP1*, *COLEC12*, *MAP3K7IP1*, *TCL1A*, *CD1C*, *CD80 *and *DOK3*, including two of the genes originally described as EBV-induced (*CCR7 *and *TRAF1*). In all cases, bisulfite pyrosequencing of these genes confirmed an at least two-fold significant decrease for the aforementioned genes (Figure 2a; Additional file 2).

### Hypomethylation associated with EBV-mediated transformation of RBLs to LCLs occurs in association with proliferation and does not involve active demethylation mechanisms

Our results indicated that the transformation of resting B cells to proliferating B lymphoblastoid cells is associated with gene promoter hypomethylation. Loss of methylation may occur as a result of the defective maintenance of DNA methylation as DNA replication and subsequent cell divisions start or, alternatively, as an active mechanism. To discriminate between these two possibilities, we first performed bisulfite genomic pyrosequencing of the genes previously analyzed in samples generated over time, including points at 1, 3 and 24 hours, before cell proliferation had been initiated [19], and LCLs after 1 and 2 weeks. To this end, we used a form of EBV that infects B cells very efficiently [20] and in which around 90% of B cells express EBNA2 (Figure 2b). The results showed that significant demethylation only occurred after cell divisions have started to take place (Figure 2c), although the coincidence in time with replication does not necessarily mean that hypomethylation takes place through a passive mechanism.

A variety of factors are known to be involved in active demethylation. Recent studies have drawn attention towards a family of enzymes, the Tet proteins, that convert 5-methylcytosine (5mC) to 5-hydroxymethylcytosine (5hmC) and other modified forms of cytosine. 5hmC may represent intermediates in the process leading to active DNA demethylation [21,22]. In addition, activation-induced deaminase (AID) participates in active demethylation in a two-step process, whereby 5mC is first deaminated by AID to thymine, followed by G/T mismatch-specific thymine DNA glycosylase (TDG)-mediated excision repair [22,23]. This process could potentially take place on 5mC, although some studies have shown that hydroxymethylation may target the methylcytosine residues that are going to be demethylated through this process [22]. Analysis of the 5hmC levels at different times in several genes that become hypomethylated during RBL to LCL transformation showed neither a significant presence of 5hmC on the CpGs that undergo hypomethylation nor any changes during this process (Figure 2d). We did not see any significant change in the expression levels of Tet proteins (not shown) during this process. We also investigated the potential involvement of AID in hypomethylation. AID is overexpressed during EBV-mediated conversion of RBLs to LCLs. RT-PCR and western blot analysis of AID revealed an increased expression of AID during this process (Figure 2e). Recent ChIP-seq data have also served to identify direct AID targets in B cells [24]. Comparison of these data with our own hypomethylation data showed that 71 out of 256 genes overlapped with AID binding sites (Figure 2f), although a chi-square test indicated that there is no significant enrichment. We also generated a retroviral inducible system for AID in a B cell line to test whether forced expression of this factor could lead to hypomethylation in the aforementioned genes. Bisulfite pyrosequencing of the same CpG sites of the previously validated genes before and after expression of AID showed no differences in methylation (not shown).

Given that EBV-mediated transformation and B cell activation share common pathways [25], we also performed bisulfite pyrosequencing of these genes in B cells activated/stimulated with IL4 and CD40L to test the EBV-associated specificity of the observed DNA demethylation changes. Under our conditions we achieved similar levels of both cell activation, measured by cytometry analysis of CD86 surface marker, and proliferation, measured by bromodeoxyuridine (BrdU) incorporation (Figure 2g, top) in both types of stimulation of B cells. However, no changes in the DNA methylation levels of these genes were observed during CD40L/IL4-stimulation of B cells (see examples in Figure 2g, bottom) even after 1 week, suggesting that methylation changes associated with EBV-mediated transformation to LCLs are independent of B cell activation.

### Hypomethylated genes are enriched for binding of NF-κB and other lymphocyte-specific transcription factors

Our analysis of the dynamics of DNA demethylation suggests that this process occurs in a replication-associated manner, since changes only occurred once cell divisions had started and did not involve changes in 5hmC or the action of AID. In this context, hypomethylation could potentially associate with genomic sites that are less efficient in maintaining methylation during DNA replication, perhaps in regions that are associated with active transcription in the context of B cell function.

**Figure 3:**
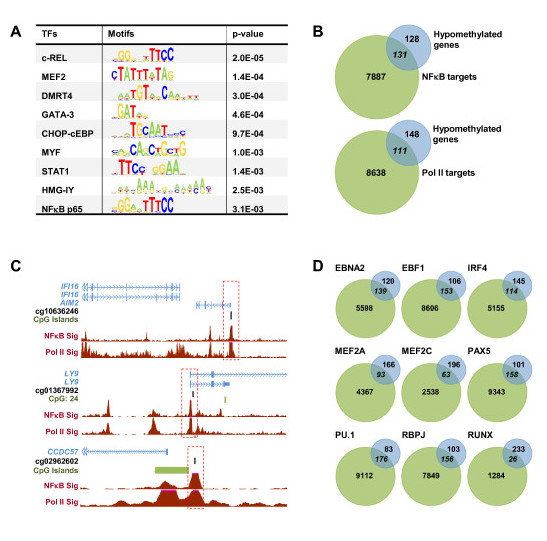
**Association of factors with hypomethylation during RBL to LCL conversion**. **(a) **Significant enrichment of predicted transcription factors (TFs; TRANSFAC motif) in hypomethylated regions. The 500-bp region around the center of significantly hypomethylated CpG sites was tested. Color intensity in the heatmap cells represents FDR *P*-values. Redder and yellower colors indicate greater and lower significance, respectively; gray indicates insignificance. **(b) **Venn diagram showing overlap of NF-κB targets from ChIPseq (considering targets that are also within the hypomethylated list) and hypomethylated genes (the 131 overlapping genes are listed in Additional file 4). **(c) **Three examples showing NF-κB p65 binding to the region neighboring the hypomethylated CpGs. The binding motif location is presented as a horizontal green bar. Below, NF-κB p65 and RNA polymerase II (Pol II) binding from GM12878 ChIP-seq data are shown. **(d) **Venn diagrams showing overlap between hypomethylated genes and transcription factors EBF1, IRF4, MEF2A, MEF2C, PAX5, PU.1, RBPJ, RUNX and EBNA2.

Previous studies have shown that EBV transcription factor EBNA2 enhances and exploits the RBL transcription program by binding to a variety of B cell transcription factor sites [4]. We wondered whether hypomethylation is taking place at genomic sites bound by specific transcription factors, perhaps associated with active transcription in B cells. To address this, we investigated the possible enrichment of transcription factor motifs from the TRANSFAC database in a region of 500 bp around the CpG sites in which hypomethylation had been detected in our study. Significant enrichment of a small set of transcription factors was observed. Remarkably, significant enrichment of the binding motifs of two subunits of the NF-κB complex, specifically c-REL (9.3%, *P*-value = 2.0 × 10^-5^) and p65/RELA (6.3%, *P*-value = 3.1 × 10^-3^) (Figure 3a) was observed. NF-κB is known to be involved in the survival of LCLs and latency III-regulated cell gene expression [26]. We also found enrichment of additional hematopoietic transcription factor binding motifs in this 500-bp window around hypomethylated CpG sites, such as GATA3, STAT1 and the MEF2 family (Figure 3a).

To evaluate the extent to which hypomethylated genes correlate with NF-κB occupancy, we used our methylation data and ChIP-seq data for NF-κB p65. Enriched NF-κB p65 peaks from a ChIP-seq study in lymphoblastoid cells [27] were annotated to the nearest Ensembl gene build (version 54) [28] using the Bioconductor package ChIPpeakAnno [29]. All the targets from all four replicates were uniquely combined. Overlap of hypomethylated genes and NF-κB p65 targets were represented in a Venn diagram and the significance of overlap was determined by a standard chi-square test, written in the syntax of the R statistical program (Figure 3b).

We found that among the entire set of unique direct NF-κB p65 targets, 131 genes were shared with our list of 256 hypomethylated genes, that is, 51% of our hypomethylated genes were directly associated with the NF-κB p65 subunit (Figure 3b). Examples of the detailed binding of NF-κB p65 to these hypomethylated regions are shown in Figure 3c. Additional ChIP-seq data for other transcription factors were also used to investigate the overlap with hypomethylated genes. Specifically, we used ChIP-seq data from EBF1, IRF4, MEF2A, MEF2C, PAX5 and PU1 obtained from GM12878 cells (LCLs) from the ENCODE project (Figure 3D). Remarkably, significant enrichment was obtained for the binding of EBF1, IRF4 and MEF2C (*P*-value < 0.05). EBF1 is a transcription factor that is critical for both B lymphopoiesis and B cell function [30]. IRF4 is as a crucial transcription factor in the generation of functionally competent plasma B cells [31]. Finally, transcription factor Mef2c is required for B cell proliferation and survival after antigen receptor stimulation [32].

#### chipseq

Our results suggested an association between genes that are regulated by B cell-specific transcription factors, particularly some associated with B cell activation and proliferation, and those that become hypomethylated. It is possible that during transformation of B cells to proliferative lymphoblasts, cells are less efficient in maintaining DNA methylation at actively transcribed sites, perhaps due to a lower presence of DNA methyltransferases (DNMTs), which are preferably bound to heterochromatic regions [33,34]. One could argue that in quiescent B cells there is a tendency for inactive genes to become methylated, and when the cells are activated to grow and proliferate, genes that become active lose methylation. This possibility was partially discarded by analyzing the methylation levels in CD40L/IL4-activated B cells (Figure 2g). Also, the analysis of bone marrow CD19+ cells, where a high proportion of B cells are proliferating [35], showed no methylation differences with respect to peripheral blood CD19+ cells, which are quiescent (Additional file 3). It could also be possible that the finding of lower methylation levels in proliferating B cells was due to the specific infection by EBV of a B cell subpopulation with lower levels of methylation. To address that, we pyrosequenced several genes in different B cell subpopulations present in peripheral blood, including naïve B cells, as well as unswitched and switched memory B cells. No differences in methylation were observed with respect to total CD19+ cells from peripheral blood or RBLs and they all displayed higher methylation than LCLs (Additional file 3).

### Several key genes are overexpressed is association with hypomethylation during EBV-mediated transformation to LCLs

Our findings suggest that many of the genes undergoing hypomethylation are actively transcribed in B cells, and therefore loss of methylation may not directly affect their expression levels. However, it is important to determine whether hypomethylation is also associated with overexpression of genes that may confer an advantage to proliferating B lymphoblasts.

**Figure 4:**
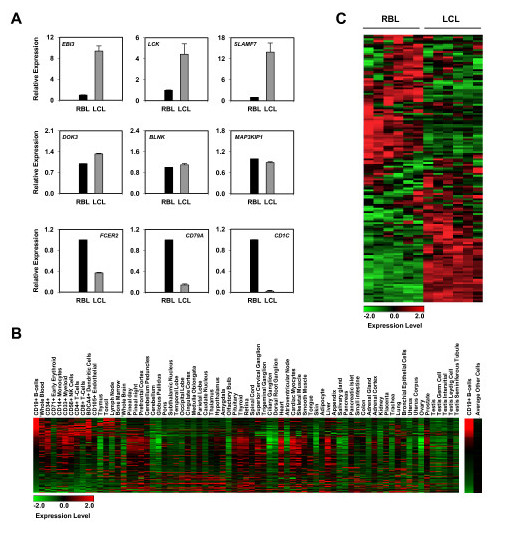
**Expression analysis of genes undergoing hypomethylation during RBL to LCL conversion**. **(a) **Expression analysis of selected genes in RBLs and matching LCLs. Error bars represent the standard deviation obtained from six independent measurements. **(b) **Heatmap showing the relative expression of the 256 genes hypomethylated in B cells with respect to other cell and tissue types. Expression data from Affymetrix mRNA expression analysis with 73 normal human tissues [36]. **(c) **Heatmap showing the relative expression of the 256 genes hypomethylated in B cells with respect to LCLs. Expression data obtained from GSE26212 [16].

To address this matter, we performed quantitative PCR of the selected genes in the set of six paired RBL and LCL samples (Figure 4a). Loss of methylation was in some cases associated with an increase in gene expression. This happens for instance for all *bona fide *EBV-induced genes (EBI), like *EBI1*, *EBI2 *and *EBI3 *(Figure 4a). Individual analysis also showed increase in mRNA levels for other genes, although in other cases this correlation with hypomethylation did not occur, and no changes or even a decrease in expression was observed, although the fold change was relatively low (see central and bottom panels in Figure 4a). This is not surprising, given that the DNA methylation levels of some of the genes undergoing hypomethylation in this process were already relatively low in resting B cells. As mentioned above, many of these hypomethylated genes are key factors in B cell function (*CD19*, *CD79a*, *BLNK*, and so on) and they are expected to be highly expressed in resting B cells. To test whether RNA polymerase II (Pol II) is associated with these genes we overlapped our data with the ChIP-seq data and found that at least 111 of them were Pol II-bound genes (Figure 3b). Examples of the detailed binding of Pol II to these hypomethylated regions are shown in Figure 3c.

We tested whether our 256 hypomethylated genes were generally highly expressed in B cells relative to other cell types. Thus, we compared normalized Affymetrix mRNA expression data of 73 normal human tissues [36]. Comparison of expression of the 256 hypomethylated genes indicated that around 28% of them were more highly expressed in B cells than the average expression level of the 73 other tissue types (Figure 4b), reinforcing our findings on the relevance of the B cell context in the genes that undergo hypomethylation.

For a general comparison in the context of transformation of RBLs to LCLs, we compared the expression profiles of the 256 genes hypomethylated in B and LCLs (available at the Gene Expression Omnibus (GEO) database under series accession number GSE26212) [16]. This revealed that around 41% of the genes displayed high levels of expression in RBLs (Figure 4c). However, around 59% of the remaining genes accomplished significant increase in gene expression (Figure 4b; Additional file 4), correlating with the over two-fold reduction in DNA methylation. Remarkably, the list of genes that become hypomethylated and are overexpressed includes several key genes in RBL to LCL conversion (Additional file 4). For instance, *EBI3 *undergoes an approximately five-fold change in methylation and becomes overexpressed. This gene is a subunit of the heterodimeric cytokine IL27, known to be regulated through NF-κB activation [37] and to induce B cell proliferation, which is stronger in naïve than in memory B cells [38]. Another example is LTA, or lymphotoxin alpha, a member of the tumor necrosis factor family, and an autocrine growth factor induced upon binding of NF-κB [39]. Other examples in our list include genes like *SLAM1 *and *SLAMF7*, two members of the 'signaling lymphocyte activation molecule' (SLAM) family, also implicated in B cell proliferation [40].

### Pharmacologically induced DNA demethylation enhances proliferation during RBL to LCL transformation

**Figure 5:**
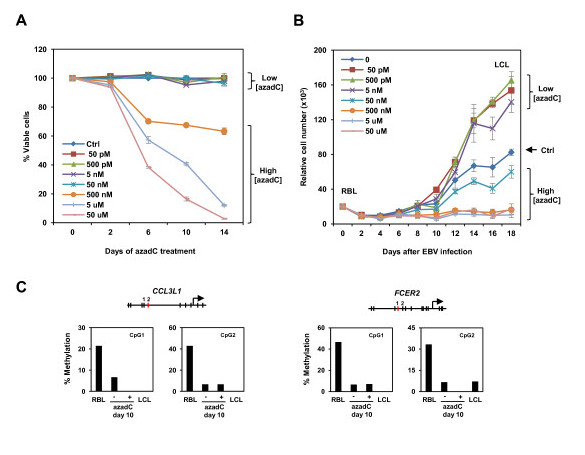
**Effects of 5azadC in RBL to LCL conversion**. **(a) **Influence of 5azadC treatments in viability of LCLs determined by flow cytometry based on propidium ioidide fluorescence. **(b) **Influence of 5azadC treatments in proliferation, as determined by MTT assays, during RBL to LCL conversion. **(c) **Bisulfite sequencing of two example genes, where methylation levels of two CpG sites is shown (the one represented in the array is highlighted in red). Methylation analysis was done in RBL cells at 10 days after EBV infection in the absence and presence of azadC, and in LCLs.

Our analysis revealed that a high proportion of the genes that undergo hypomethylation during RBL to LCL transformation become overexpressed and that several of them are important for the transformation on continuous proliferating B lymphoblasts. To determine whether hypomethylation has any effect on the efficiency of transformation, we investigated whether pharmacologically induced hypomethylation influences RBL to LCL transformation. We tested the proliferation rate in RBLs infected with EBV and incubated in the presence of increasing concentrations with the demethylating agent 5-azadeoxycytidine (azadC) at concentrations between 50 pM and 50 μM. MTT assays showed that mock-treated cells started to proliferate around day 6 after infection with EBV (Figure 5a). Cell viability and proliferation rate decreased at very high doses of azadC, consistent with the toxicity properties of azadC at high doses, which we tested on cultured LCLs (Figure 5a). However, at low azadC concentrations (50 pM, 500 pM and 5 nM), where viability of cells is comparable to that of control cells (Figure 5a), a significant increase in cell proliferation was observed after day 8 (Figure 5b). This increase in proliferation in the presence of azadC was not observed when established LCLs were used as a control in proliferation experiments in the presence of azadC, suggesting that the effect of azadC is associated with the transformation of RBLs to proliferating B cells. Bisulfite genomic analysis of several of the target genes confirmed the loss of methylation at the studied CpG sites upon treatment with 5azadC at low concentrations (Figure 5c). In fact, we observed that azadC-treated cells had lower levels of methylation than control cells at day 10 (Figure 5c). Analysis of viral promoters showed that these were unmethylated in both free viral particles and in LCLs, ruling out the possibility that demethylation at EBV promoters and overexpression of EBV-encoded genes are responsible for more efficient transformation of RBLs to LCLs.

## Discussion

Our results provide evidence that EBV-mediated transformation of RBLs to LCLs results in the demethylation of a cluster of around 250 genes. Conversely, no significant hypermethylation is observed. Hypomethylation has been described in various immune disease-related contexts, including ICF (immunodeficiency, centromere instability and facial anomalies) syndrome [41] and autoimmune diseases [8]. In most cases, disease-associated hypomethylation occurs at repetitive sequences, including Alu elements [42]. However, in RBL to LCL transformation, hypomethylation takes place near the transcription start sites of around 250 genes, and no apparent hypomethylation occurs at the major type of repetitive elements. Lack of hypomethylation in repetitive sequences is consistent with the minimal changes in genomic stability associated with EBV-associated lymphoproliferation.

Given that hypomethylation occurs in association with the acquisition of proliferation, it is likely to be related to the decreased efficiency in DNA methylation maintenance as cells start to divide. The lack of evidence for active demethylation mechanisms during this process also supports the above possibility. The transition of a differentiated resting cell to a proliferative status would require the participation of DNMTs to maintain the DNA methylation profiles throughout replication/division cycles. Various lines of evidence indicate that DNMTs are associated with repetitive sequences [33,34] and that heterochromatic regions act as a reservoir for DNMTs [33]. This would explain why active regions, which are less rich in DNMTs, lose methylation in the transformation from resting to proliferative B cells, whereas in ICF syndrome, which is characterized by mutations in DNMT3B, hypomethylation takes place in heterochromatic regions, which act as reservoirs of DNMTs.

In fact, analysis of the regions associated with hypomethylation during RBL to LCL transformation shows enrichment of highly expressed genes and target sites of transcription factors that are expressed in B cells or are specifically activated during EBV infection, such as factors in the NF-κB pathway [26,43]. The modulation of the NF-κB signaling by EBV is not only important for viral infection, but also contributes to the development of malignant neoplasia [43]. In addition, our results indicate that hypomethylated genes are regulated by B cell-specific factors like EBF1, IRF4 and MEF2C, the last two of which are implicated in B cell activation and proliferation processes. We also observed that many of the genes that undergo hypomethylation, like *CD19*, *CD79a*, *BLNK*, *BLK *and *LCK*, are highly expressed in B cells. This suggests that active regions are either more accessible to putative demethylating machinery or are less efficient at maintaining methylation as proliferation starts. Given the lack of demethylation events before proliferation begins, and that we were unable to detect links between any of the proposed mechanisms associated with active demethylation, we favor the occurrence of a replication-coupled mechanism of demethylation, due to the inefficient maintenance of DNA methylation as replication is initiated. Similar to studies of the EBNA2 binding sites [4] in which EBV exploits intrinsic B cell transcription programs, hypomethylation takes place preferentially in genes that are expressed. Comparison of the lists of significantly hypomethylated genes and the presence of transcription factor binding motifs and occupancy reinforced this notion. Data from different sources suggest that transcriptionally active regions are devoid of DNMTs, making the maintenance of DNA methylation less efficient. On the other hand, since the main functional consequence of promoter hypomethylation would potentially be an increase in transcription, it is likely that demethylation contributes to overexpression of some of these genes. In this sense, we have identified several key genes that undergo both hypomethylation and overexpression during the conversion of RBLs to LCLs. These include genes like *EBI3 *[37,38], a subunit of the cytokine IL27, *LTA *[39] and members of the *SLAM *family [40] among others that are known to contribute to lymphocyte proliferation (Additional file 4).

In addition, demethylation of genes that already have high expression levels in the first set of infected cells might also lock its transcriptional status, and therefore would reinforce the B cell phenotype, and might be key to enable infection of more B cells. EBV initially infects oropharynx epithelial cells, establishing a lytic replication that spreads to some B cells in nearby lymphoid tissues. Those expand as EBV establishes a type III latency growth-transforming infection of B cells that elicit a strong T-cell-specific response. Maintaining a B cell phenotype would favor recirculation and generalized expansion to B cell areas in secondary lymphoid organs where surviving B cells downregulate EBV antigen expression and establish long-term silent latency [44].

The functional relevance of demethylation in this process is highlighted by the observation that treatment of RBLs with azadC at low sub-toxic concentrations enhances RBL to LCL transformation. Since the EBV genome is virtually unmethylated in free viral particles, demethylation at EBV promoters and overexpression of EBV-encoded genes can be ruled out as responsible for more efficient transformation of RBLs to LCLs. Our findings on the enhanced cell proliferation concomitantly associated with the presence of low sub-toxic amounts of azadC suggests that initial DNA methylation changes may cooperate in the efficiency of this process.

Although type III latency lymphoblastoid cells do not necessarily correspond to initial steps of lymphomagenesis, they both share enhanced proliferation [45]. It is likely that hypomethylation occurs in early steps towards to lymphomagenesis, when enhanced proliferation starts. In other hematological neoplasias, like in acute myeoloid leukemia, *DNMT3A *mutations are highly recurrent [46], and it has been proposed that these mutations in an enzyme responsible for the establishment of DNA methylation are an early event of clonal evolution [47]. In B-cell chronic lymphocytic leukemia, increased levels of *TCL1 *result in inhibition of DNA methylation [48]. Inhibition of methylation is proposed to be a common oncogenic mechanism in leukemogenesis [48]. These findings highlight the relevance of our study as a potential early mechanism in this group of tumors.

## Conclusions

Our study of DNA methylation changes in EBV-mediated transformation of RBLs to LCLs reveals that only promoter hypomethylation occurs during this process. Neither significant hypermethylation nor methylation changes at repetitive elements are observed. Hypomethylation takes place only when proliferation has started, and the analysis of putative elements of the active demethylation machinery does not indicate their implication during this process. Most genes undergoing hypomethylation are active and display the presence of NF-κB p65 and other B-cell-specific transcription factors. Since DNMTs tend to associate with heterochromatic regions, it is likely that maintenance of DNA methylation is less efficient in transcriptionally active regions as cells start to proliferate triggered by EBV; however, this process does not occur during CD40L/IL4-stimulation of B cells where no changes are observed. Our results show that hypomethylation is associated with further upregulation of gene expression of many of these genes. Also, pharmacologically induced demethylation increases B cell transformation efficiency and proliferation. Collectively, our data indicate that the B cell transcription machinery is associated with the subset of genes that undergo hypomethylation. The finding that relevant genes in EBV-mediated transformation of B cells are further upregulated indicates a key role of this mechanism. Hypomethylation has been proposed to play a role in early stages of hematological malignancies, including other B cell malignancies. Our data reinforce the notion of the role of hypomethylation in early lymphomagenesis and shed light on underlying mechanisms.

## Materials and methods

### Ethics statement

Human samples (blood) used in this study come from anonymous blood donors and were obtained from the Catalan blood donation center (Banc de Sang i Teixits). Since the samples are anonymous, no informed consent is therefore required. The protocol used to transform with EBV B cells obtained from these anonymous donors was approved by the Committee of Biosecurity of IDIBELL (CBS) on 5 May 2011 and the Ethics Committee of the University Hospital of Bellvitge (CEIC) on 28 May 2011.

### Subjects and sample preparation

Buffy-coats from anonymous blood donors were obtained from the Catalan blood donation center (Banc de Sang i Teixits). Viable peripheral blood mononuclear cells were isolated using LymphoprepTM density gradient centrifugation. Resting B cells were isolated by positive selection using CD19 MicroBeads (Miltenyi Biotec, Bergisch Gladbach, Germany), or by depletion using a B Cell Isolation Kit (Miltenyi Biotec). Isolated B cells were immortalized with the supernatant of the EBV producer cell line B95.8 for the methylation studies and with the 2089 EBV made from 293 cells carrying a recombinant B95.8 EBV genome [20] for expression analysis.

### DNA methylation profiling using universal bead arrays

Infinium Methylation Assay (Illumina, Inc., San Diego, CA, USA) was used to analyze DNA methylation. The HumanMethylation27 panel allows researchers to interrogate 27,578 highly informative CpG sites per sample at single-nucleotide resolution. This panel targets CpG sites located within the proximal promoter regions of transcription start sites of 14,475 consensus coding sequencing (CCDS) in the NCBI Database (Genome Build 36). In addition, 254 assays cover 110 microRNA promoters. On average, two assays were selected per CCDS gene and from 3 to 20 CpG sites for > 200 cancer-related and imprinted genes. Bisulfite conversion of DNA samples was done using the EZ DNA methylation kit (Zymo Research, Orange, CA, USA). After bisulfite treatment, the remaining assay steps were identical to those of the Infimium Methylation Assay, using reagents and conditions supplied and recommended by Illumina. Two technical replicates of each bisulfite-converted sample were run. The results were all in close agreement and were averaged for subsequent analysis. The array hybridization was conducted under a temperature gradient program, and arrays were imaged using a BeadArray Reader (Illumina Inc.). The image processing and intensity data extraction software and procedures were those described by Bibikova and colleagues [49]. Each methylation data point was represented as a combination of the Cy3 and Cy5 fluorescent intensities from the M (methylated) and U (unmethylated) alleles. Background intensity computed from a set of negative controls was subtracted from each data point.

### Detection of differentially methylated genes from the methylation array and functional analysis

A *t*-test was carried out to identify probes differentially methylated between primary B cells relative to their counterparts obtained in the LCL. *P*-values were corrected for multiple testing using the method proposed by Benjamini and Hochberg [50] to control the FDR. Genes showing a FDR adjusted *P*-value < 0.05 and a minimum mean methylation FC of two were considered to be differentially methylated. Functional annotation of hypomethylated genes was based on GO (Consortium, 2000), as extracted from EnsEMBL [28] and the KEGG pathway database [51]. Accordingly, all genes were classified into three ontologies, based on their involvement in biological processes, molecular functions and cellular components. We took only the GO/pathway categories that had at least ten annotated genes. We used GiTools for enrichment analysis and heatmap generation [52]. Resulting *P*-values were adjusted for multiple testing using Benjamini and Hochberg's FDR method [50]. An FDR cutoff of 0.25 was used for selection of enriched terms. The relationships between the DNA methylation data (from standard bisulfite sequencing and/or quantitative bisulfite pyrosequencing) and the age and sex of the individuals were evaluated by the Pearson chi-square test.

### Analysis of gene promoter methylation: bisulfite sequencing and pyrosequencing

CpG island DNA methylation status was determined by sequencing bisulfite-modified genomic DNA. Bisulfite modification of genomic DNA was carried out as described by Herman *et al. *[53]. To validate the DNA methylation data obtained by the Infinium methylation assay, bisulfite pyrosequencing was performed according to standard protocols and evaluated with the Pyro Q-CpG 1.0.9 program (Biotage, Uppsala, Sweden). Primer sequences, product lengths and annealing temperatures used in the bisulfite sequencing and bisulfite pyrosequencing PCR reactions are shown in Additional file 5. Raw data for bisulfite sequencing of all samples is presented in Additional file 6.

### Amplification of unmethylated Alus (AUMA)

DNA digestion with SmaI enzyme and ligation to the linker were performed as described elsewhere [18]. The product was purified using the GFX Kit (Amersham Biosciences, Uppsala Sweden) and eluted in 250 μl of sterile water. A chimeric primer comprising the complementary linker sequence (ATTCGCAAAGCTCTGA), the cut SmaI site (GGG) and three additional nucleotides homologous to the Alu consensus sequence were used to enrich for Alu sequences: AUMA-TTC (ATTCGCAAAGCTCTGAGGGTTC). Single primers were used for each PCR reaction. Products were resolved on denaturing sequencing gels. Bands were visualized by silver staining the gels. Faint bands with inconsistent display due to small variations in gel electrophoresis resolution were not considered. Band reproducibility was assessed with the analysis of PCR duplicates. AUMA fingerprints were visually checked for methylation differences between bands in different samples. Based on these premises, a given band was scored according to three possible behaviors: hypomethylation (increased intensity), hypermethylation (decreased intensity) and no change (no substantial difference between samples). Only those bands showing clear changes in their fingerprint intensities were considered to represent methylation changes.

### Quantitative RT-PCR expression analyses

We reverse-transcribed total RNA extracted with TRIzol (Invitrogen), using a Transcriptor First Strand cDNA Synthesis Kit from Roche Diagnostics (Indianapolis, Indiana, USA). Quantitative real-time PCR analysis was performed in a PCR Real Time LightCycler 480 (Roche) with Sybr green. Primer sequences are shown in Additional file 5.

### Analysis of transcription factor binding

Possible enrichment of transcription factor motif in the 500- to 1,000-bp region around the center of the hypomethylated probes and all other probes were predicted with the STORM algorithm [54], assuming *P*-value cutoffs of 0.00002 and 0.00001, respectively, using position frequency matrices (PFMs) from the TRANSFAC database (Professional version, release 2009.4) [55]. Enrichment analysis of predicted transcription factors in the probes of significant hypomethylated probes (*n *= 421) were conducted using GiTools [52]. We calculated a two-tailed *P*-value, and a finally adjusted FDR *P*-value (with 0.25 cutoff) was considered in establishing statistical significance.

We downloaded EBF1, IRF4, MEF2A, MEF2C, PAX5 and PU1 binding ChIPseq data for the GM12878 cell line from the ENCODE project [56]. Original hg19 genomic co-ordinates were converted to hg18 using the USCS 'liftover' tool. RUNX ChIPseq target data were obtained from the study of Hollenhorst and colleagues [57] (Jurkat cell line, GEO Database ID: GSE17954). RBPJ and EBNA2 taken from a 2011 study of Zhao *et al. *[4] (GEO database ID: GSE29498). In this case, mapped data were analyzed using the MACS (version 1.3.7.1) [58] pipeline to call peaks. In all cases, peaks were annotated to the nearest EnsEMBL [28] gene (version 54) using the Bioconductor package ChIPpeakAnno [29].

### Expression analysis of hypomethylated genes in various tissue types

Expression data on RBL and LCL from the study of Caliskan and colleagues [16] (GEO accession GSE26210) were used to determine the relative expression of hypomethylated genes in these two cell types. Probes were annotated to Refseq genes and when more than one probe was present for the same gene, they were averaged. Also, all replicates on same sample were averaged. Expression data were normalized by median-centering the expression value of each gene across all the samples and dividing the value by the standard deviation. These normalized values were delineated in a color-coded heatmap using GiTools [52].

Normalized mRNA expression of data of 73 normal human tissues was downloaded from the BioGPS database [36] and analyzed similarly.

### Cell proliferation and viability assays

In cell proliferation analysis, different dilutions of cells were plated and cultured at 37°C in 5% CO_2_/95% O_2 _for 20 days. AzadC (Sigma, St. Louis, MO, USA) was used in serial dilutions between 50 pM and 50 μM and refreshed on day 4 of treatment. Every 2 days, cells were fixed and stained with MTT and incubated for 4 hours at 37°C. The reaction was stopped with 50% N,N-dimethylformamide, 30% SDS, 2.5% glacial acetic acid and 2.5% acid chloride 1 N, and incubated overnight at 37°C in 5% CO_2_/95% O_2_. Cell quantities were determined by measuring the optical density at 560 nm. All assays were performed in triplicate. Cell viability was determined by the incorporation of propidium iodide in dead cells measured by flow cytometry.

### CD40L activation of B cells

B cells were cultured at 1.5 × 106 cells/ml and activated with 50 ng/ml of M.CD40L (Enzo Life Sciences, Lausen, Switzerland) and 50 ng/ml of IL4 (Gentaur, Kampenhout, Belgium). The percentage of activated RBLs was determined by CD86 expression measured by flow cytometry and proliferating B cells were detected by measuring BrdU incorporation.

### Use of a B-cell-based inducible system to test AID activity

Jiyoye B cells with inducible expression of AID were generated using the Retro-X TM Tet-ON^® ^Advanced Inducible Expression System (Clontech, Saint-Germain-en-Laye, France). This system works through the sequential infection of the RetroX-Tet-ON advanced vector and the pRetroX-Tight-Pur vector. Carboxy-terminal hemagglutinin (HA)-tagged human AID was cloned in the pRetroX-Tight-Pur vector. The stable doubly infected cell line was selected with Geneticin (1 mg/ml) and Puromycin (0.3 μg/ml). AID expression was induced by the addition of doxycycline (500 ng/ml) for 24 hours. Nuclear export was inhibited by the addition of leptomycin B (10 ng/ml) for 2 hours.

## Data access

Methylation array data for this publication have been deposited in NCBI's GEO and is accessible through GEO series accession number GSE41957 [59].

## Abbreviations

5hmC: 5-hydroxymethylcytosine; 5mC: 5-methylcytosine; AID: activation-induced deaminase; AUMA: amplification of unmethylated Alu repeats; azadC: 5-azadeoxycytidine; BrdU: bromodeoxyuridine; ChIP: chromatin immunoprecipitation; DNMT: DNA methyltransferase; EBV: Epstein-Barr virus; FC: fold change; FDR: false discovery rate; GEO: Gene Expression Omnibus; GO: Gene Ontology; LCL: lymphoblastoid cell line; NF: nuclear factor; RBL: resting B lymphocyte; Pol II: RNA polymerase II.

## Competing interests

The authors declare that they have no competing interests.

## Authors' contributions

HH, CS-L, JR-U, VR-C, BJ, CM and MP performed experiments, AI, FA-S, AF, MF performed analysis, H-JD contributed with key tools, ME, EL-G, NL-B contributed with key analytic tools and interpreted data, and EB designed the project and wrote the paper. All authors read and approved the final manuscript.

## Supplementary Material

Additional file 1 **Hypomethylated genes in RBL to LCL transformation for a FDR adjusted *P*-value < 0.05 and fold change (FC) ≥ 2 from bead array analysis**. Click here for file

Additional file 2 **Comparison of the methylation levels (in percentage) for selected genes from the bead arrays and calculated after bisulfite pyrosequencing**. Click here for file

Additional file 3 **A comparison of the DNA methylation levels of selected genes in different B cell types**. Bisulfite pyrosequencing was performed for the genes selected from experiments with methylation arrays. The analysis includes bone marrow (BM) CD19+ cells, naïve B cells, unswitched (US) memory B cells and switched (S) memory cells. Also peripheral blood resting B cells (RBLs) and corresponding lymphoblastoid B cells (LCLs) are included. Click here for file

Additional file 4 **Expression changes between those undergoing hypomethylation during the conversion between resting B cells and lymphoblastoid cells**. Click here for file

Additional file 5 **Primer sequences**. Click here for file

Additional file 6 **Individual raw data corresponding to bisulfite pyrosequencing presented in **Figure 2. Click here for file

## References

[B1] Saha A Robertson ES Epstein-Barr virus-associated B-cell lymphomas: pathogenesis and clinical outcomes. Clin Cancer Res 2011 17 3056 3063 10.1158/1078-0432.CCR-10-2578 21372216 PMC4287361

[B2] Pender MP Infection of autoreactive B lymphocytes with EBV, causing chronic autoimmune diseases. Trends Immunol 2003 24 584 588 10.1016/j.it.2003.09.005 14596882

[B3] Cohen JI Wang F Mannick J Kieff E Epstein-Barr virus nuclear protein 2 is a key determinant of lymphocyte transformation. Proc Natl Acad Sci USA 1989 86 9558 9562 10.1073/pnas.86.23.9558 2556717 PMC298536

[B4] Zhao B Zou J Wang H Johannsen E Peng CW Quackenbush J Mar JC Morton CC Freedman ML Blacklow SC Aster JC Bernstein BE Kieff E Epstein-Barr virus exploits intrinsic B-lymphocyte transcription programs to achieve immortal cell growth. Proc Natl Acad Sci USA 2011 108 14902 14907 10.1073/pnas.1108892108 21746931 PMC3169132

[B5] Deaton AM Bird A CpG islands and the regulation of transcription. Genes Dev 2011 25 1010 1022 10.1101/gad.2037511 21576262 PMC3093116

[B6] Esteller M Gaidano G Goodman SN Zagonel V Capello D Botto B Rossi D Gloghini A Vitolo U Carbone A Baylin SB Herman JG Hypermethylation of the DNA repair gene O(6)-methylguanine DNA methyltransferase and survival of patients with diffuse large B-cell lymphoma. J Natl Cancer Inst 2002 94 26 32 10.1093/jnci/94.1.26 11773279

[B7] Ammerpohl O Haake A Pellissery S Giefing M Richter J Balint B Kulis M Le J Bibikova M Drexler HG Seifert M Shaknovic R Korn B Küppers R Martín-Subero JI Siebert R Array-based DNA methylation analysis in classical Hodgkin lymphoma reveals new insights into the mechanisms underlying silencing of B cell-specific genes. Leukemia 2011 26 185 188 21818115 10.1038/leu.2011.194

[B8] Javierre BM Fernandez AF Richter J Al-Shahrour F Martin-Subero JI Rodriguez-Ubreva J Berdasco M Fraga MF O'Hanlon TP Rider LG Jacinto FV Lopez-Longo FJ Dopazo J Forn M Peinado MA Carreño L Sawalha AH Harley JB Siebert R Esteller M Miller FW Ballestar E Changes in the pattern of DNA methylation associate with twin discordance in systemic lupus erythematosus. Genome Res 2010 20 170 179 10.1101/gr.100289.109 20028698 PMC2813473

[B9] Ji H Ehrlich LI Seita J Murakami P Doi A Lindau P Lee H Aryee MJ Irizarry RA Kim K Rossi DJ Inlay MA Serwold T Karsunky H Ho L Daley GQ Weissman IL Feinberg AP Comprehensive methylome map of lineage commitment from haematopoietic progenitors. Nature 2010 467 338 342 10.1038/nature09367 20720541 PMC2956609

[B10] Fernandez AF Rosales C Lopez-Nieva P Graña O Ballestar E Ropero S Espada J Melo SA Lujambio A Fraga MF Pino I Javierre B Carmona FJ Acquadro F Steenbergen RD Snijders PJ Meijer CJ Pineau P Dejean A Lloveras B Capella G Quer J Buti M Esteban JI Allende H Rodriguez-Frias F Castellsague X Minarovits J Ponce J Capello D The dynamic DNA methylomes of double-stranded DNA viruses associated with human cancer. Genome Res 2009 19 438 451 19208682 10.1101/gr.083550.108 PMC2661803

[B11] Schaefer BC Strominger JL Speck SH Host-cell-determined methylation of specific Epstein-Barr virus promoters regulates the choice between distinct viral latency programs. Mol Cell Biol 1997 17 364 377 8972217 10.1128/mcb.17.1.364 PMC231761

[B12] Bakos A Banati F Koroknai A Takacs M Salamon D Minarovits-Kormuta S Schwarzmann F Wolf H Niller HH Minarovits J High-resolution analysis of CpG methylation and in vivo protein-DNA interactions at the alternative Epstein-Barr virus latency promoters Qp and Cp in the nasopharyngeal carcinoma cell line C666-1. Virus Genes 2007 35 195 202 10.1007/s11262-007-0095-y 17510783

[B13] Fejer G Koroknai A Banati F Gyory I Salamon D Wolf H Niller HH Minarovits J Latency type-specific distribution of epigenetic marks at the alternative promoters Cp and Qp of Epstein-Barr virus. J Gen Virol 2008 89 1364 1370 10.1099/vir.0.83594-0 18474551

[B14] Hutchings IA Tierney RJ Kelly GL Stylianou J Rickinson AB Bell AI Methylation status of the Epstein-Barr virus (EBV) BamHI W latent cycle promoter and promoter activity: analysis with novel EBV-positive Burkitt and lymphoblastoid cell lines. J Virol 2006 80 10700 10711 10.1128/JVI.01204-06 16920819 PMC1641762

[B15] Paschos K Smith P Anderton E Middeldorp JM White RE Allday MJ Epstein-barr virus latency in B cells leads to epigenetic repression and CpG methylation of the tumour suppressor gene Bim. PLoS Pathog 2009 5 e1000492 10.1371/journal.ppat.1000492 19557159 PMC2695769

[B16] Caliskan M Cusanovich DA Ober C Gilad Y The effects of EBV transformation on gene expression levels and methylation profiles. Hum Mol Genet 2011 20 1643 1652 10.1093/hmg/ddr041 21289059 PMC3063990

[B17] Leonard S Wei W Anderton J Vockerodt M Rowe M Murray PG Woodman CB Epigenetic and transcriptional changes which follow Epstein-Barr virus infection of germinal center B cells and their relevance to the pathogenesis of Hodgkin's lymphoma. J Virol 2011 85 9568 9577 10.1128/JVI.00468-11 21752916 PMC3165764

[B18] Rodriguez J Vives L Jorda M Morales C Munoz M Vendrell E Peinado MA Genome-wide tracking of unmethylated DNA Alu repeats in normal and cancer cells. Nucleic Acids Res 2008 36 770 784 18084025 10.1093/nar/gkm1105 PMC2241897

[B19] Shannon-Lowe C Baldwin G Feederle R Bell A Rickinson A Delecluse HJ Epstein-Barr virus-induced B-cell transformation: quantitating events from virus binding to cell outgrowth. J Gen Virol 2005 86 3009 3019 10.1099/vir.0.81153-0 16227222

[B20] Delecluse HJ Hilsendegen T Pich D Zeidler R Hammerschmidt W Propagation and recovery of intact, infectious Epstein-Barr virus from prokaryotic to human cells. Proc Natl Acad Sci USA 1998 95 8245 8250 10.1073/pnas.95.14.8245 9653172 PMC20961

[B21] Wu H Zhang Y Mechanisms and functions of Tet protein-mediated 5-methylcytosine oxidation. Genes Dev 2011 25 2436 2452 10.1101/gad.179184.111 22156206 PMC3243055

[B22] Cortellino S Xu J Sannai M Moore R Caretti E Cigliano A Le Coz M Devarajan K Wessels A Soprano D Abramowitz LK Bartolomei MS Rambow F Bassi MR Bruno T Fanciulli M Renner C Klein-Szanto AJ Matsumoto Y Kobi D Davidson I Alberti C Larue L Bellacosa A Thymine DNA glycosylase is essential for active DNA demethylation by linked deamination-base excision repair. Cell 2011 146 67 79 10.1016/j.cell.2011.06.020 21722948 PMC3230223

[B23] Rai K Huggins IJ James SR Karpf AR Jones DA Cairns BR DNA demethylation in zebrafish involves the coupling of a deaminase, a glycosylase, and gadd45. Cell 2008 135 1201 1212 10.1016/j.cell.2008.11.042 19109892 PMC2629358

[B24] Yamane A Resch W Kuo N Kuchen S Li Z Sun HW Robbiani DF McBride K Nussenzweig MC Casellas R Deep-sequencing identification of the genomic targets of the cytidine deaminase AID and its cofactor RPA in B lymphocytes. Nat Immunol 2011 12 62 69 10.1038/ni.1964 21113164 PMC3005028

[B25] Thorley-Lawson DA Epstein-Barr virus: exploiting the immune system. Nat Rev Immunol 2001 1 75 82 10.1038/35095584 11905817

[B26] Cahir-McFarland ED Carter K Rosenwald A Giltnane JM Henrickson SE Staudt LM Kieff E Role of NF-kappa B in cell survival and transcription of latent membrane protein 1-expressing or Epstein-Barr virus latency III-infected cells. J Virol 2004 78 4108 4119 10.1128/JVI.78.8.4108-4119.2004 15047827 PMC374271

[B27] Kasowski M Grubert F Heffelfinger C Hariharan M Asabere A Waszak SM Habegger L Rozowsky J Shi M Urban AE Hong MY Karczewski KJ Huber W Weissman SM Gerstein MB Korbel JO Snyder M Variation in transcription factor binding among humans. Science 2010 328 232 235 10.1126/science.1183621 20299548 PMC2938768

[B28] Hubbard TJ Aken BL Beal K Ballester B Caccamo M Chen Y Clarke L Coates G Cunningham F Cutts T Down T Dyer SC Fitzgerald S Fernandez-Banet J Graf S Haider S Hammond M Herrero J Holland R Howe K Howe K Johnson N Kahari A Keefe D Kokocinski F Kulesha E Lawson D Longden I Melsopp C Megy K Ensembl 2007. Nucleic Acids Res 2007 35 D610 617 10.1093/nar/gkl996 17148474 PMC1761443

[B29] Zhu LJ Gazin C Lawson ND Pages H Lin SM Lapointe DS Green MR ChIPpeakAnno: a Bioconductor package to annotate ChIP-seq and ChIP-chip data. BMC Bioinformatics 2010 11 237 10.1186/1471-2105-11-237 20459804 PMC3098059

[B30] Hagman J Ramirez J Lukin K B lymphocyte lineage specification, commitment and epigenetic control of transcription by early B cell factor 1. Curr Top Microbiol Immunol 2012 356 17 38 21735360 10.1007/82_2011_139 PMC3925327

[B31] Klein U Casola S Cattoretti G Shen Q Lia M Mo T Ludwig T Rajewsky K Dalla-Favera R Transcription factor IRF4 controls plasma cell differentiation and class-switch recombination. Nat Immunol 2006 7 773 782 10.1038/ni1357 16767092

[B32] Wilker PR Kohyama M Sandau MM Albring JC Nakagawa O Schwarz JJ Murphy KM Transcription factor Mef2c is required for B cell proliferation and survival after antigen receptor stimulation. Nat Immunol 2008 9 603 612 18438409 10.1038/ni.1609 PMC2518613

[B33] Kashiwagi K Nimura K Ura K Kaneda Y DNA methyltransferase 3b preferentially associates with condensed chromatin. Nucleic Acids Res 2011 39 874 888 10.1093/nar/gkq870 20923784 PMC3035464

[B34] Choi SH Heo K Byun HM An W Lu W Yang AS Identification of preferential target sites for human DNA methyltransferases. Nucleic Acids Res 2011 39 104 118 10.1093/nar/gkq774 20841325 PMC3017615

[B35] McGinnes K Letarte M Paige CJ B-lineage colonies from normal, human bone marrow are initiated by B cells and their progenitors. Blood 1991 77 961 970 1704806

[B36] Su AI Wiltshire T Batalov S Lapp H Ching KA Block D Zhang J Soden R Hayakawa M Kreiman G Cooke MP Walker JR Hogenesch JB A gene atlas of the mouse and human protein-encoding transcriptomes. Proc Natl Acad Sci USA 2004 101 6062 6067 10.1073/pnas.0400782101 15075390 PMC395923

[B37] Wirtz S Becker C Fantini MC Nieuwenhuis EE Tubbe I Galle PR Schild HJ Birkenbach M Blumberg RS Neurath MF EBV-induced gene 3 transcription is induced by TLR signaling in primary dendritic cells via NF-kappa B activation. J Immunol 2005 174 2814 2824 15728491 10.4049/jimmunol.174.5.2814

[B38] Larousserie F Charlot P Bardel E Froger J Kastelein RA Devergne O Differential effects of IL-27 on human B cell subsets. J Immunol 2006 176 5890 5897 16670296 10.4049/jimmunol.176.10.5890

[B39] Thompson MP Aggarwal BB Shishodia S Estrov Z Kurzrock R Autocrine lymphotoxin production in Epstein-Barr virus-immortalized B cells: induction via NF-kappaB activation mediated by EBV-derived latent membrane protein 1. Leukemia 2003 17 2196 2201 10.1038/sj.leu.2403130 14523478

[B40] Sayos J Wu C Morra M Wang N Zhang X Allen D van Schaik S Notarangelo L Geha R Roncarolo MG Oettgen H De Vries JE Aversa G Terhorst C The X-linked lymphoproliferative-disease gene product SAP regulates signals induced through the co-receptor SLAM. Nature 1998 395 462 469 10.1038/26683 9774102

[B41] Kondo T Bobek MP Kuick R Lamb B Zhu X Narayan A Bourc'his D Viegas-Pequignot E Ehrlich M Hanash SM Whole-genome methylation scan in ICF syndrome: hypomethylation of non-satellite DNA repeats D4Z4 and NBL2. Hum Mol Genet 2000 9 597 604 10.1093/hmg/9.4.597 10699183

[B42] Weisenberger DJ Campan M Long TI Kim M Woods C Fiala E Ehrlich M Laird PW Analysis of repetitive element DNA methylation by MethyLight. Nucleic Acids Res 2005 33 6823 6836 10.1093/nar/gki987 16326863 PMC1301596

[B43] de Oliveira DE Ballon G Cesarman E NF-kappaB signaling modulation by EBV and KSHV. Trends Microbiol 2010 18 248 257 10.1016/j.tim.2010.04.001 20452220

[B44] Young LS Rickinson AB Epstein-Barr virus: 40 years on. Nat Rev Cancer 2004 4 757 768 10.1038/nrc1452 15510157

[B45] Kuppers R Klein U Hansmann ML Rajewsky K Cellular origin of human B-cell lymphomas. N Engl J Med 1999 341 1520 1529 10.1056/NEJM199911113412007 10559454

[B46] Ley TJ Ding L Walter MJ McLellan MD Lamprecht T Larson DE Kandoth C Payton JE Baty J Welch J Harris CC Lichti CF Townsend RR Fulton RS Dooling DJ Koboldt DC Schmidt H Zhang Q Osborne JR Lin L O'Laughlin M McMichael JF Delehaunty KD McGrath SD Fulton LA Magrini VJ Vickery TL Hundal J Cook LL Conyers JJ DNMT3A mutations in acute myeloid leukemia. N Engl J Med 2010 363 2424 2433 10.1056/NEJMoa1005143 21067377 PMC3201818

[B47] Shih AH Abdel-Wahab O Patel JP Levine RL The role of mutations in epigenetic regulators in myeloid malignancies. Nat Rev Cancer 2012 12 599 612 10.1038/nrc3343 22898539

[B48] Palamarchuk A Yan PS Zanesi N Wang L Rodrigues B Murphy M Balatti V Bottoni A Nazaryan N Alder H Rassenti L Kipps TJ Freitas M Croce CM Pekarsky Y Tcl1 protein functions as an inhibitor of de novo DNA methylation in B-cell chronic lymphocytic leukemia (CLL). Proc Natl Acad Sci USA 2012 109 2555 2560 10.1073/pnas.1200003109 22308499 PMC3289317

[B49] Bibikova M Chudin E Wu B Zhou L Garcia EW Liu Y Shin S Plaia TW Auerbach JM Arking DE Gonzalez R Crook J Davidson B Schulz TC Robins A Khanna A Sartipy P Hyllner J Vanguri P Savant-Bhonsale S Smith AK Chakravarti A Maitra A Rao M Barker DL Loring JF Fan JB Human embryonic stem cells have a unique epigenetic signature. Genome Res 2006 16 1075 1083 10.1101/gr.5319906 16899657 PMC1557765

[B50] Benjamini Y Hochberg Y Controlling the false discovery rate: a practical and powerful approach to multiple testing. J R Stat Soc B 1995 57 289 300

[B51] Kanehisa M Goto S KEGG: kyoto encyclopedia of genes and genomes. Nucleic Acids Res 2000 28 27 30 10.1093/nar/28.1.27 10592173 PMC102409

[B52] Perez-Llamas C Lopez-Bigas N Gitools: analysis and visualisation of genomic data using interactive heat-maps. PLoS One 2011 6 e19541 10.1371/journal.pone.0019541 21602921 PMC3094337

[B53] Herman JG Graff JR Myohanen S Nelkin BD Baylin SB Methylation-specific PCR: a novel PCR assay for methylation status of CpG islands. Proc Natl Acad Sci USA 1996 93 9821 9826 10.1073/pnas.93.18.9821 8790415 PMC38513

[B54] Schones DE Smith AD Zhang MQ Statistical significance of cis-regulatory modules. BMC Bioinformatics 2007 8 19 10.1186/1471-2105-8-19 17241466 PMC1796902

[B55] Matys V Fricke E Geffers R Gössling E Haubrock M Hehl R Hornischer K Karas D Kel AE Kel-Margoulis OV Kloos DU Land S Lewicki-Potapov B Michael H Münch R Reuter I Rotert S Saxel H Scheer M Thiele S Wingender E TRANSFAC: transcriptional regulation, from patterns to profiles. Nucleic Acids Res 2003 31 374 378 10.1093/nar/gkg108 12520026 PMC165555

[B56] Transcription Factor Binding Sites by ChIP-seq from ENCODE/HAIB. http://genome.ucsc.edu/cgi-bin/hgFileUi?db=hg19&g=wgEncodeHaibTfbs

[B57] Hollenhorst PC Chandler KJ Poulsen RL Johnson WE Speck NA Graves BJ DNA specificity determinants associate with distinct transcription factor functions. PLoS Genet 2009 5 e1000778 10.1371/journal.pgen.1000778 20019798 PMC2787013

[B58] Zhang Y Liu T Meyer CA Eeckhoute J Johnson DS Bernstein BE Nusbaum C Myers RM Brown M Li W Liu XS Model-based analysis of ChIP-Seq (MACS). Genome Biol 2008 9 R137 10.1186/gb-2008-9-9-r137 18798982 PMC2592715

[B59] GEO Series GSE41957. http://www.ncbi.nlm.nih.gov/geo/query/acc.cgi?acc=GSE41957

