# Supplementary material for: The B cell transcription program mediates hypomethylation and overexpression of key genes in Epstein-Barr virus-associated proliferative conversion
Source: Genome Biol. 2013 Jan 15;14(1):R3. doi: 10.1186/gb-2013-14-1-r3 (PMC3663113; doi:10.1186/gb-2013-14-1-r3)
Supplement: Additional file 1 — Hypomethylated genes in RBL to LCL transformation for a FDR adjusted P-value < 0.05 and fold change (FC) ≥ 2 from bead array analysis. [file gb-2013-14-1-r3-S1.DOC]

**Additional File 1**

**Hypomethylated genes in RBL to LCL transformation for which FDR adjusted p value < 0.05 and fold change (FC) ≥ 2 from bead array analysis**

| **Gene Symbol** | **RBL Mean** | **RBL SD** | **LCL Mean** | **LCL SD** | **Feature P** | **FDR** | **FC** |
| --- | --- | --- | --- | --- | --- | --- | --- |
| CCL3L1 | 0.21 | 0.05 | 0.04 | 0.02 | 9.95E-005 | 0.01 | 5.63 |
| KSR1 | 0.24 | 0.07 | 0.04 | 0.02 | 0 | 0.02 | 5.4 |
| ARRDC4 | 0.07 | 0.02 | 0.01 | 0.01 | 6.31E-004 | 0.01 | 5.25 |
| EBI3 | 0.14 | 0.05 | 0.03 | 0 | 0 | 0.03 | 5.12 |
| HIST1H2BO | 0.26 | 0.07 | 0.05 | 0.02 | 4.21E-004 | 0.01 | 5.04 |
| VDAC3 | 0.11 | 0.03 | 0.02 | 0.01 | 6.66E-004 | 0.01 | 4.52 |
| ABI3 | 0.47 | 0.1 | 0.11 | 0.08 | 6.94E-005 | 0.01 | 4.42 |
| RGS20 | 0.22 | 0.05 | 0.05 | 0.03 | 1.67E-004 | 0.01 | 4.36 |
| L3MBTL4 | 0.14 | 0.04 | 0.03 | 0.01 | 5.93E-004 | 0.01 | 4.28 |
| FCER2 | 0.1 | 0.03 | 0.02 | 0.01 | 5.34E-004 | 0.01 | 4.22 |
| AIM2 | 0.27 | 0.1 | 0.06 | 0.05 | 0 | 0.03 | 4.15 |
| S100A7 | 0.14 | 0.04 | 0.03 | 0.02 | 8.87E-004 | 0.02 | 4.1 |
| SLAMF7 | 0.28 | 0.07 | 0.07 | 0.02 | 4.80E-004 | 0.01 | 4.03 |
| LY96 | 0.26 | 0.08 | 0.07 | 0.03 | 0 | 0.02 | 4 |
| PXDN | 0.16 | 0.04 | 0.04 | 0.01 | 7.66E-004 | 0.01 | 3.99 |
| FGD2 | 0.09 | 0.04 | 0.02 | 0.01 | 0.01 | 0.05 | 3.97 |
| SIX2 | 0.1 | 0.02 | 0.03 | 0.01 | 3.76E-006 | 0 | 3.96 |
| C9orf89 | 0.19 | 0.05 | 0.05 | 0.03 | 1.89E-004 | 0.01 | 3.92 |
| SP140 | 0.16 | 0.05 | 0.04 | 0.01 | 8.83E-004 | 0.02 | 3.91 |
| C8orf44 | 0.34 | 0.08 | 0.09 | 0.03 | 2.09E-004 | 0.01 | 3.85 |
| EBI2 | 0.32 | 0.12 | 0.08 | 0.08 | 0 | 0.04 | 3.8 |
| CYR61 | 0.07 | 0.02 | 0.02 | 0 | 4.53E-004 | 0.01 | 3.8 |
| EPB41L3 | 0.33 | 0.06 | 0.09 | 0.09 | 3.27E-004 | 0.01 | 3.79 |
| G6PC | 0.3 | 0.09 | 0.08 | 0.03 | 9.84E-004 | 0.02 | 3.78 |
| PSMAL | 0.45 | 0.1 | 0.12 | 0.06 | 1.37E-004 | 0.01 | 3.76 |
| ANGPTL6 | 0.23 | 0.04 | 0.06 | 0.01 | 9.82E-005 | 0.01 | 3.72 |
| BLNK | 0.19 | 0.04 | 0.05 | 0.02 | 5.45E-005 | 0.01 | 3.68 |
| IL17E | 0.31 | 0.09 | 0.09 | 0.03 | 6.75E-004 | 0.01 | 3.67 |
| IRS2 | 0.06 | 0.01 | 0.02 | 0 | 2.95E-004 | 0.01 | 3.67 |
| LCE2A | 0.14 | 0.03 | 0.04 | 0.01 | 2.81E-004 | 0.01 | 3.62 |
| PPAN | 0.25 | 0.04 | 0.07 | 0.04 | 3.00E-005 | 0.01 | 3.62 |
| ACSM2 | 0.28 | 0.1 | 0.08 | 0.04 | 0 | 0.03 | 3.53 |
| ZBED2 | 0.18 | 0.03 | 0.05 | 0.01 | 5.64E-006 | 0 | 3.52 |
| APOL6 | 0.14 | 0.04 | 0.04 | 0.01 | 6.65E-004 | 0.01 | 3.46 |
| COL5A1 | 0.1 | 0.02 | 0.03 | 0.01 | 4.03E-005 | 0.01 | 3.43 |
| SPINK5 | 0.63 | 0.12 | 0.18 | 0.06 | 6.38E-005 | 0.01 | 3.43 |
| PFTK1 | 0.18 | 0.05 | 0.05 | 0.02 | 0 | 0.02 | 3.43 |
| WBSCR17 | 0.16 | 0.05 | 0.05 | 0.01 | 0 | 0.03 | 3.38 |
| LRRN6C | 0.45 | 0.15 | 0.13 | 0.06 | 0 | 0.03 | 3.36 |
| CD72 | 0.16 | 0.03 | 0.05 | 0.01 | 1.40E-004 | 0.01 | 3.35 |
| LTA | 0.2 | 0.04 | 0.06 | 0.01 | 1.88E-004 | 0.01 | 3.31 |
| MLLT11 | 0.12 | 0.03 | 0.04 | 0.01 | 2.62E-004 | 0.01 | 3.29 |
| NID2 | 0.09 | 0.02 | 0.03 | 0.01 | 5.19E-004 | 0.01 | 3.28 |
| TNFAIP8 | 0.22 | 0.04 | 0.07 | 0.04 | 7.73E-005 | 0.01 | 3.28 |
| C11orf10 | 0.4 | 0.07 | 0.12 | 0.03 | 5.39E-005 | 0.01 | 3.25 |
| LGI2 | 0.05 | 0.02 | 0.02 | 0 | 0.01 | 0.05 | 3.22 |
| AHR | 0.18 | 0.06 | 0.05 | 0.03 | 0 | 0.02 | 3.22 |
| POLD4 | 0.04 | 0.01 | 0.01 | 0 | 0 | 0.02 | 3.22 |
| CCL18 | 0.54 | 0.1 | 0.17 | 0.07 | 5.28E-005 | 0.01 | 3.2 |
| GAL3ST3 | 0.1 | 0.02 | 0.03 | 0.02 | 2.00E-004 | 0.01 | 3.18 |
| CNIH3 | 0.08 | 0.02 | 0.02 | 0.01 | 0 | 0.03 | 3.13 |
| C7orf19 | 0.05 | 0.01 | 0.02 | 0.01 | 3.17E-004 | 0.01 | 3.13 |
| CCDC57 | 0.2 | 0.06 | 0.06 | 0.03 | 8.84E-004 | 0.02 | 3.13 |
| SFT2D3 | 0.14 | 0.03 | 0.04 | 0.02 | 7.74E-005 | 0.01 | 3.11 |
| TRAF1 | 0.25 | 0.06 | 0.08 | 0.02 | 5.08E-004 | 0.01 | 3.11 |
| PDGFC | 0.12 | 0.02 | 0.04 | 0.01 | 1.14E-004 | 0.01 | 3.05 |
| IPF1 | 0.08 | 0.01 | 0.03 | 0.01 | 2.05E-008 | 1.49E-004 | 3.05 |
| TRIM48 | 0.23 | 0.07 | 0.07 | 0.03 | 0 | 0.03 | 3.02 |
| PDCD1 | 0.32 | 0.06 | 0.1 | 0.08 | 4.72E-004 | 0.01 | 3.01 |
| PLA2G7 | 0.06 | 0.01 | 0.02 | 0.01 | 1.58E-004 | 0.01 | 3 |
| LCP1 | 0.13 | 0.02 | 0.04 | 0.03 | 2.99E-004 | 0.01 | 3 |
| TAP1 | 0.2 | 0.07 | 0.07 | 0.03 | 0 | 0.04 | 2.98 |
| LAMA1 | 0.18 | 0.03 | 0.06 | 0.02 | 3.77E-005 | 0.01 | 2.95 |
| PRAF1 | 0.11 | 0.02 | 0.04 | 0.01 | 4.13E-005 | 0.01 | 2.92 |
| KITLG | 0.13 | 0.02 | 0.04 | 0.01 | 2.08E-005 | 0 | 2.91 |
| HIST1H2BJ | 0.1 | 0.03 | 0.04 | 0.01 | 0 | 0.02 | 2.86 |
| LILRB1 | 0.34 | 0.08 | 0.12 | 0.05 | 3.81E-004 | 0.01 | 2.84 |
| FLJ23235 | 0.21 | 0.03 | 0.07 | 0.03 | 7.97E-006 | 0 | 2.83 |
| CD19 | 0.1 | 0.03 | 0.03 | 0.01 | 0 | 0.04 | 2.82 |
| RASGRF2 | 0.1 | 0.02 | 0.03 | 0.02 | 7.12E-004 | 0.01 | 2.81 |
| MGAT5 | 0.43 | 0.07 | 0.15 | 0.09 | 2.43E-004 | 0.01 | 2.81 |
| CYYR1 | 0.15 | 0.03 | 0.06 | 0.03 | 2.99E-004 | 0.01 | 2.81 |
| S100A2 | 0.16 | 0.03 | 0.06 | 0.01 | 2.51E-004 | 0.01 | 2.8 |
| TM6SF1 | 0.07 | 0.02 | 0.03 | 0.01 | 0 | 0.04 | 2.79 |
| C20orf58 | 0.13 | 0.03 | 0.05 | 0.01 | 4.03E-004 | 0.01 | 2.79 |
| NME3 | 0.14 | 0.03 | 0.05 | 0.03 | 4.78E-004 | 0.01 | 2.78 |
| IL21 | 0.33 | 0.11 | 0.12 | 0.05 | 0 | 0.03 | 2.77 |
| NCF1 | 0.07 | 0.02 | 0.03 | 0 | 0 | 0.02 | 2.74 |
| MCFP | 0.33 | 0.06 | 0.12 | 0.06 | 1.02E-004 | 0.01 | 2.74 |
| MYO3A | 0.13 | 0.02 | 0.05 | 0.02 | 1.45E-005 | 0 | 2.73 |
| ADH1B | 0.28 | 0.06 | 0.1 | 0.05 | 2.10E-004 | 0.01 | 2.72 |
| CDH11 | 0.22 | 0.03 | 0.08 | 0.03 | 3.20E-005 | 0.01 | 2.71 |
| PELO | 0.15 | 0.03 | 0.05 | 0.03 | 7.21E-004 | 0.01 | 2.69 |
| SCARF2 | 0.09 | 0.02 | 0.03 | 0 | 0 | 0.02 | 2.69 |
| DSC2 | 0.1 | 0.01 | 0.04 | 0.01 | 2.08E-007 | 6.19E-004 | 2.68 |
| DCAKD | 0.4 | 0.1 | 0.15 | 0.05 | 5.82E-004 | 0.01 | 2.68 |
| ELAVL3 | 0.12 | 0.02 | 0.04 | 0.01 | 3.39E-005 | 0.01 | 2.66 |
| PBOV1 | 0.57 | 0.1 | 0.21 | 0.08 | 6.48E-005 | 0.01 | 2.66 |
| MAPKAP1 | 0.09 | 0.02 | 0.03 | 0.01 | 1.80E-004 | 0.01 | 2.66 |
| SLC6A2 | 0.2 | 0.04 | 0.08 | 0.04 | 1.98E-004 | 0.01 | 2.65 |
| COLEC12 | 0.13 | 0.02 | 0.05 | 0.02 | 4.71E-005 | 0.01 | 2.63 |
| IGJ | 0.26 | 0.05 | 0.1 | 0.03 | 1.86E-004 | 0.01 | 2.63 |
| C20orf185 | 0.55 | 0.11 | 0.21 | 0.14 | 9.54E-004 | 0.02 | 2.62 |
| LIMD1 | 0.11 | 0.02 | 0.04 | 0.02 | 2.48E-004 | 0.01 | 2.62 |
| LOC400696 | 0.25 | 0.07 | 0.1 | 0.02 | 0 | 0.03 | 2.6 |
| FLJ39599 | 0.16 | 0.05 | 0.06 | 0.02 | 0 | 0.03 | 2.59 |
| SLC25A11 | 0.13 | 0.03 | 0.05 | 0.02 | 0 | 0.02 | 2.58 |
| SLAMF1 | 0.13 | 0.03 | 0.05 | 0.03 | 3.56E-004 | 0.01 | 2.57 |
| TRIP6 | 0.07 | 0.02 | 0.03 | 0.02 | 0 | 0.03 | 2.56 |
| SUHW2 | 0.14 | 0.04 | 0.06 | 0.04 | 0.01 | 0.05 | 2.55 |
| MAGI2 | 0.07 | 0.01 | 0.03 | 0.01 | 2.53E-004 | 0.01 | 2.54 |
| OR10J1 | 0.46 | 0.09 | 0.18 | 0.05 | 1.35E-004 | 0.01 | 2.54 |
| RIMS2 | 0.4 | 0.08 | 0.16 | 0.06 | 1.77E-004 | 0.01 | 2.53 |
| ADRA2A | 0.05 | 0.01 | 0.02 | 0.01 | 4.70E-005 | 0.01 | 2.52 |
| CEACAM4 | 0.23 | 0.06 | 0.09 | 0.04 | 0 | 0.02 | 2.51 |
| PLEK | 0.08 | 0.01 | 0.03 | 0.01 | 6.62E-005 | 0.01 | 2.5 |
| ARHGEF10 | 0.09 | 0.02 | 0.03 | 0.01 | 0 | 0.02 | 2.49 |
| EVL | 0.14 | 0.03 | 0.06 | 0.02 | 4.93E-004 | 0.01 | 2.49 |
| GPRC5C | 0.09 | 0.02 | 0.04 | 0.01 | 6.74E-004 | 0.01 | 2.47 |
| KRTAP13-2 | 0.16 | 0.05 | 0.07 | 0.02 | 0 | 0.03 | 2.47 |
| LY9 | 0.15 | 0.04 | 0.06 | 0.04 | 0 | 0.04 | 2.46 |
| C12orf36 | 0.52 | 0.08 | 0.21 | 0.04 | 6.08E-005 | 0.01 | 2.46 |
| HS3ST1 | 0.06 | 0.01 | 0.02 | 0 | 1.23E-005 | 0 | 2.46 |
| MIPOL1 | 0.14 | 0.01 | 0.06 | 0.01 | 5.96E-007 | 9.58E-004 | 2.45 |
| HRH3 | 0.06 | 0.01 | 0.02 | 0.01 | 6.94E-006 | 0 | 2.45 |
| TMEM87A | 0.18 | 0.03 | 0.07 | 0.01 | 1.46E-004 | 0.01 | 2.44 |
| MAP3K7IP1 | 0.26 | 0.04 | 0.11 | 0.02 | 1.34E-004 | 0.01 | 2.44 |
| EML3 | 0.36 | 0.03 | 0.15 | 0.05 | 1.79E-005 | 0 | 2.43 |
| CCR6 | 0.1 | 0.02 | 0.04 | 0.02 | 1.90E-004 | 0.01 | 2.41 |
| SH3GL3 | 0.09 | 0.01 | 0.04 | 0.02 | 2.76E-004 | 0.01 | 2.4 |
| PPP1R1A | 0.05 | 0.01 | 0.02 | 0.01 | 4.36E-004 | 0.01 | 2.4 |
| BLK | 0.08 | 0.02 | 0.03 | 0.02 | 0 | 0.03 | 2.4 |
| SNAP91 | 0.08 | 0.02 | 0.03 | 0 | 7.18E-004 | 0.01 | 2.4 |
| MTF1 | 0.1 | 0.02 | 0.04 | 0.03 | 0 | 0.04 | 2.39 |
| GPR55 | 0.5 | 0.1 | 0.21 | 0.09 | 3.54E-004 | 0.01 | 2.39 |
| MRPS15 | 0.11 | 0.03 | 0.05 | 0.03 | 0 | 0.04 | 2.38 |
| BCL2L14 | 0.31 | 0.08 | 0.13 | 0.08 | 0 | 0.04 | 2.38 |
| C12orf43 | 0.05 | 0.01 | 0.02 | 0.01 | 0 | 0.03 | 2.37 |
| C1R | 0.11 | 0.02 | 0.05 | 0.01 | 2.67E-004 | 0.01 | 2.37 |
| SPRR1B | 0.41 | 0.09 | 0.17 | 0.05 | 4.91E-004 | 0.01 | 2.36 |
| TCBA1 | 0.03 | 0 | 0.01 | 0 | 2.03E-004 | 0.01 | 2.35 |
| B4GALT4 | 0.11 | 0.02 | 0.05 | 0.02 | 0 | 0.02 | 2.35 |
| SOX8 | 0.05 | 0.01 | 0.02 | 0 | 5.85E-004 | 0.01 | 2.34 |
| FAM3B | 0.07 | 0.01 | 0.03 | 0.01 | 3.72E-004 | 0.01 | 2.33 |
| DHRS4L2 | 0.07 | 0.01 | 0.03 | 0.01 | 9.06E-005 | 0.01 | 2.33 |
| LOC124842 | 0.06 | 0.01 | 0.03 | 0 | 3.53E-004 | 0.01 | 2.33 |
| CCR7 | 0.17 | 0.03 | 0.08 | 0.03 | 3.15E-004 | 0.01 | 2.32 |
| RPL39L | 0.15 | 0.04 | 0.06 | 0.04 | 0 | 0.03 | 2.32 |
| KRTAP13-3 | 0.65 | 0.1 | 0.28 | 0.08 | 4.23E-005 | 0.01 | 2.31 |
| WFDC11 | 0.57 | 0.08 | 0.24 | 0.06 | 2.75E-005 | 0.01 | 2.31 |
| CPZ | 0.12 | 0.02 | 0.05 | 0.03 | 0 | 0.02 | 2.29 |
| SALL1 | 0.07 | 0 | 0.03 | 0.01 | 2.11E-006 | 0 | 2.29 |
| TNFRSF7 | 0.23 | 0.04 | 0.1 | 0.05 | 5.64E-004 | 0.01 | 2.28 |
| ACADL | 0.14 | 0.02 | 0.06 | 0.02 | 1.60E-005 | 0 | 2.28 |
| NKX6-2 | 0.22 | 0.03 | 0.1 | 0.05 | 9.45E-004 | 0.02 | 2.28 |
| AKR1C2 | 0.52 | 0.07 | 0.23 | 0.13 | 0 | 0.02 | 2.26 |
| TPM4 | 0.04 | 0.01 | 0.02 | 0.01 | 9.54E-004 | 0.02 | 2.26 |
| ISG20 | 0.18 | 0.03 | 0.08 | 0.01 | 4.62E-004 | 0.01 | 2.26 |
| RASEF | 0.08 | 0.02 | 0.03 | 0.01 | 9.04E-004 | 0.02 | 2.25 |
| ROR2 | 0.11 | 0.02 | 0.05 | 0.02 | 8.41E-005 | 0.01 | 2.25 |
| ZNF702 | 0.08 | 0.02 | 0.03 | 0.02 | 0 | 0.03 | 2.24 |
| UTF1 | 0.15 | 0.04 | 0.07 | 0.03 | 0 | 0.02 | 2.24 |
| NELF | 0.22 | 0.05 | 0.1 | 0.05 | 0 | 0.02 | 2.24 |
| PCDH7 | 0.08 | 0.01 | 0.04 | 0.01 | 1.61E-004 | 0.01 | 2.24 |
| TCL1A | 0.12 | 0.03 | 0.05 | 0.03 | 0 | 0.03 | 2.24 |
| TADA3L | 0.23 | 0.06 | 0.1 | 0.04 | 0 | 0.02 | 2.24 |
| LRIG3 | 0.13 | 0.02 | 0.06 | 0.03 | 0 | 0.02 | 2.24 |
| CFHR5 | 0.53 | 0.11 | 0.24 | 0.08 | 4.83E-004 | 0.01 | 2.24 |
| CCL3L3 | 0.06 | 0.01 | 0.03 | 0.01 | 8.24E-004 | 0.02 | 2.24 |
| KRTAP20-2 | 0.42 | 0.1 | 0.19 | 0.08 | 0 | 0.02 | 2.23 |
| HIST1H1D | 0.16 | 0.01 | 0.07 | 0.02 | 3.04E-005 | 0.01 | 2.23 |
| ECE1 | 0.04 | 0.01 | 0.02 | 0.01 | 0 | 0.03 | 2.22 |
| CUTL2 | 0.08 | 0.01 | 0.03 | 0.01 | 1.85E-004 | 0.01 | 2.22 |
| ITIH2 | 0.57 | 0.09 | 0.26 | 0.1 | 1.76E-004 | 0.01 | 2.22 |
| CD1C | 0.24 | 0.06 | 0.11 | 0.04 | 0 | 0.02 | 2.22 |
| ERRFI1 | 0.15 | 0.02 | 0.07 | 0.02 | 2.16E-005 | 0 | 2.22 |
| G0S2 | 0.13 | 0.03 | 0.06 | 0.01 | 0 | 0.02 | 2.22 |
| BPI | 0.21 | 0.03 | 0.1 | 0.02 | 2.30E-005 | 0 | 2.21 |
| CD82 | 0.14 | 0 | 0.06 | 0.03 | 0 | 0.02 | 2.21 |
| UNQ9433 | 0.13 | 0.01 | 0.06 | 0.02 | 4.41E-005 | 0.01 | 2.2 |
| RGS14 | 0.07 | 0.01 | 0.03 | 0.01 | 4.81E-004 | 0.01 | 2.2 |
| KCNJ4 | 0.09 | 0.01 | 0.04 | 0.01 | 1.31E-007 | 6.19E-004 | 2.19 |
| OSMR | 0.11 | 0.01 | 0.05 | 0.02 | 7.19E-004 | 0.01 | 2.19 |
| NETO1 | 0.11 | 0.02 | 0.05 | 0.02 | 1.87E-004 | 0.01 | 2.19 |
| SNRPD2 | 0.16 | 0.04 | 0.07 | 0.04 | 0 | 0.04 | 2.19 |
| ATPAF1 | 0.06 | 0.01 | 0.03 | 0.01 | 3.55E-006 | 0 | 2.18 |
| CLIC2 | 0.3 | 0.09 | 0.14 | 0.03 | 0.01 | 0.05 | 2.18 |
| SPACA3 | 0.49 | 0.09 | 0.22 | 0.07 | 2.72E-004 | 0.01 | 2.18 |
| GUCA1A | 0.43 | 0.11 | 0.2 | 0.06 | 0 | 0.03 | 2.17 |
| SGCZ | 0.57 | 0.08 | 0.27 | 0.11 | 3.73E-004 | 0.01 | 2.17 |
| SLC24A3 | 0.1 | 0.01 | 0.05 | 0.01 | 1.23E-004 | 0.01 | 2.17 |
| CD80 | 0.46 | 0.1 | 0.21 | 0.14 | 0.01 | 0.05 | 2.16 |
| FAT | 0.15 | 0.02 | 0.07 | 0.02 | 1.05E-005 | 0 | 2.15 |
| SLITRK5 | 0.07 | 0.01 | 0.03 | 0.01 | 5.36E-004 | 0.01 | 2.15 |
| SYT9 | 0.21 | 0.03 | 0.1 | 0.04 | 6.61E-004 | 0.01 | 2.15 |
| CD79A | 0.22 | 0.05 | 0.1 | 0.05 | 0 | 0.02 | 2.15 |
| MPO | 0.51 | 0.1 | 0.24 | 0.06 | 4.10E-004 | 0.01 | 2.15 |
| PDK4 | 0.23 | 0.04 | 0.11 | 0.05 | 5.02E-004 | 0.01 | 2.15 |
| C21orf99 | 0.19 | 0.04 | 0.09 | 0.02 | 6.19E-004 | 0.01 | 2.15 |
| IGSF21 | 0.21 | 0.04 | 0.1 | 0.04 | 6.29E-004 | 0.01 | 2.15 |
| BTBD3 | 0.22 | 0.03 | 0.1 | 0.05 | 0 | 0.02 | 2.14 |
| PRKG1 | 0.08 | 0.01 | 0.04 | 0.01 | 4.51E-005 | 0.01 | 2.14 |
| CLEC2D | 0.17 | 0.04 | 0.08 | 0.02 | 0 | 0.02 | 2.14 |
| ZNF530 | 0.11 | 0.01 | 0.05 | 0.03 | 0.01 | 0.05 | 2.13 |
| ALK | 0.07 | 0.01 | 0.03 | 0.01 | 4.26E-005 | 0.01 | 2.13 |
| CST3 | 0.07 | 0.01 | 0.03 | 0.01 | 4.66E-004 | 0.01 | 2.13 |
| SLCO2A1 | 0.05 | 0.01 | 0.02 | 0 | 3.37E-005 | 0.01 | 2.13 |
| CYP4Z1 | 0.44 | 0.08 | 0.21 | 0.13 | 0 | 0.04 | 2.13 |
| PRSS1 | 0.78 | 0.08 | 0.37 | 0.06 | 2.56E-006 | 0 | 2.13 |
| PRDM15 | 0.08 | 0.02 | 0.04 | 0.02 | 0 | 0.03 | 2.13 |
| GSH1 | 0.2 | 0.02 | 0.1 | 0.03 | 3.71E-005 | 0.01 | 2.12 |
| MS4A1 | 0.07 | 0.02 | 0.03 | 0.01 | 0 | 0.03 | 2.12 |
| SNX8 | 0.15 | 0.02 | 0.07 | 0.01 | 7.40E-005 | 0.01 | 2.11 |
| CSEN | 0.33 | 0.04 | 0.16 | 0.05 | 7.27E-005 | 0.01 | 2.11 |
| KCNK17 | 0.09 | 0.02 | 0.04 | 0.02 | 0 | 0.02 | 2.11 |
| FLJ12700 | 0.22 | 0.01 | 0.11 | 0.02 | 1.11E-006 | 0 | 2.11 |
| SOX6 | 0.42 | 0.06 | 0.2 | 0.09 | 5.79E-004 | 0.01 | 2.11 |
| KCNK1 | 0.09 | 0.01 | 0.04 | 0.01 | 1.02E-004 | 0.01 | 2.11 |
| GNA15 | 0.05 | 0.01 | 0.02 | 0.01 | 0 | 0.04 | 2.11 |
| SLCO1B1 | 0.47 | 0.1 | 0.22 | 0.09 | 0 | 0.02 | 2.1 |
| HBEGF | 0.16 | 0.01 | 0.08 | 0.02 | 2.55E-005 | 0.01 | 2.1 |
| TRIM58 | 0.04 | 0.01 | 0.02 | 0.01 | 0 | 0.05 | 2.1 |
| FLJ42486 | 0.25 | 0.04 | 0.12 | 0.06 | 0 | 0.02 | 2.1 |
| SPINT1 | 0.16 | 0.03 | 0.08 | 0.02 | 4.83E-004 | 0.01 | 2.1 |
| IBRDC1 | 0.52 | 0.11 | 0.25 | 0.1 | 0 | 0.02 | 2.09 |
| LCK | 0.09 | 0.01 | 0.04 | 0.02 | 7.61E-004 | 0.01 | 2.09 |
| NHN1 | 0.08 | 0.02 | 0.04 | 0.01 | 8.62E-004 | 0.02 | 2.09 |
| WNT3A | 0.08 | 0.01 | 0.04 | 0.01 | 5.88E-005 | 0.01 | 2.08 |
| EDNRA | 0.39 | 0.06 | 0.19 | 0.07 | 2.33E-004 | 0.01 | 2.08 |
| KPNA1 | 0.06 | 0.01 | 0.03 | 0.01 | 6.98E-004 | 0.01 | 2.08 |
| TEX15 | 0.38 | 0.08 | 0.19 | 0.06 | 0 | 0.02 | 2.07 |
| NCF4 | 0.13 | 0.02 | 0.06 | 0.01 | 7.26E-004 | 0.01 | 2.07 |
| NGFB | 0.12 | 0.03 | 0.06 | 0.02 | 0 | 0.02 | 2.07 |
| SPRR1A | 0.48 | 0.07 | 0.24 | 0.09 | 2.85E-004 | 0.01 | 2.06 |
| GPR123 | 0.31 | 0.06 | 0.15 | 0.04 | 3.50E-004 | 0.01 | 2.06 |
| SEMA7A | 0.13 | 0.03 | 0.06 | 0.04 | 0.01 | 0.05 | 2.05 |
| DSG4 | 0.55 | 0.08 | 0.27 | 0.05 | 9.89E-005 | 0.01 | 2.05 |
| EBF3 | 0.11 | 0.01 | 0.05 | 0.01 | 1.85E-005 | 0 | 2.05 |
| CASQ2 | 0.63 | 0.08 | 0.31 | 0.02 | 1.05E-004 | 0.01 | 2.05 |
| BMPR1B | 0.09 | 0.01 | 0.05 | 0.02 | 5.23E-004 | 0.01 | 2.05 |
| GPR15 | 0.72 | 0.05 | 0.35 | 0.14 | 8.69E-004 | 0.02 | 2.05 |
| PTPRM | 0.06 | 0.01 | 0.03 | 0.01 | 1.48E-005 | 0 | 2.05 |
| FLJ40235 | 0.77 | 0.09 | 0.38 | 0.09 | 1.68E-005 | 0 | 2.04 |
| OR2S2 | 0.5 | 0.07 | 0.24 | 0.1 | 5.84E-004 | 0.01 | 2.04 |
| LOC132321 | 0.48 | 0.09 | 0.24 | 0.13 | 0 | 0.04 | 2.04 |
| RASSF2 | 0.1 | 0.01 | 0.05 | 0.02 | 2.78E-004 | 0.01 | 2.04 |
| IL1F7 | 0.69 | 0.08 | 0.34 | 0.05 | 9.68E-006 | 0 | 2.04 |
| PNPLA4 | 0.13 | 0.02 | 0.07 | 0.03 | 0 | 0.02 | 2.04 |
| DSG1 | 0.5 | 0.07 | 0.25 | 0.06 | 4.63E-005 | 0.01 | 2.04 |
| EFNA2 | 0.07 | 0.01 | 0.04 | 0.01 | 6.15E-004 | 0.01 | 2.03 |
| AMID | 0.08 | 0.01 | 0.04 | 0.01 | 7.89E-006 | 0 | 2.03 |
| LGI1 | 0.73 | 0.08 | 0.36 | 0.11 | 7.85E-005 | 0.01 | 2.03 |
| CSMD1 | 0.27 | 0.03 | 0.13 | 0.03 | 4.15E-005 | 0.01 | 2.03 |
| UNC5C | 0.12 | 0.01 | 0.06 | 0.01 | 8.84E-006 | 0 | 2.02 |
| AKR1C1 | 0.43 | 0.11 | 0.21 | 0.07 | 0 | 0.04 | 2.02 |
| AJAP1 | 0.11 | 0.02 | 0.05 | 0.02 | 4.42E-004 | 0.01 | 2.02 |
| FAM9B | 0.79 | 0.06 | 0.39 | 0.19 | 0 | 0.03 | 2.02 |
| IL8RA | 0.53 | 0.09 | 0.26 | 0.05 | 2.23E-004 | 0.01 | 2.02 |
| CYP26B1 | 0.15 | 0.01 | 0.08 | 0.01 | 2.06E-006 | 0 | 2.02 |
| CHRNA4 | 0.13 | 0.02 | 0.07 | 0.01 | 4.47E-005 | 0.01 | 2.01 |
| PCDH17 | 0.08 | 0.01 | 0.04 | 0.01 | 4.12E-004 | 0.01 | 2.01 |
| DOK3 | 0.09 | 0.02 | 0.05 | 0.01 | 0 | 0.05 | 2.01 |
| PTPN14 | 0.06 | 0.01 | 0.03 | 0.01 | 0 | 0.03 | 2 |
| TRY1 | 0.64 | 0.08 | 0.32 | 0.14 | 0 | 0.02 | 2 |
| HHCM | 0.14 | 0.04 | 0.07 | 0.02 | 0.01 | 0.05 | 2 |
| CTNNA2 | 0.13 | 0.01 | 0.06 | 0.03 | 0 | 0.02 | 2 |
