# Supplementary material for: The B cell transcription program mediates hypomethylation and overexpression of key genes in Epstein-Barr virus-associated proliferative conversion
Source: Genome Biol. 2013 Jan 15;14(1):R3. doi: 10.1186/gb-2013-14-1-r3 (PMC3663113; doi:10.1186/gb-2013-14-1-r3)
Supplement: Additional file 2 — Comparison of the methylation levels (in percentage) for selected genes from the bead arrays and calculated after bisulfite pyrosequencing. [file gb-2013-14-1-r3-S2.DOC]

**Additional file 2**

**Comparison of the methylation levels (in percentage) for selected genes from the bead arrays and calculated after bisulfite pyrosequencing**

| **METHYLATION BEAD ARRAY** | | | | | |
| --- | --- | --- | --- | --- | --- |
| **GENES** | **%METH. RBL** | **SD** | **%METH. LCL** | **SD** | **FC** |
| **CCL3L1** | 21,3 | 4,89 | 3,79 | 2,14 | 5,63 |
| **FCER2** | 10,4 | 2,63 | 2,47 | 0,57 | 4,22 |
| **SLAMF7** | 27,8 | 7,15 | 6,91 | 2,29 | 4,03 |
| **BLNK** | 18,7 | 3,73 | 5,08 | 2,18 | 3,68 |
| **IL25** | 31,2 | 8,59 | 8,51 | 3,46 | 3,67 |
| **IRS2** | 6,2 | 1,37 | 1,69 | 0,38 | 3,67 |
| **TRAF1** | 25,2 | 5,95 | 8,11 | 2,01 | 3,11 |
| **TAP1** | 19,6 | 6,56 | 6,59 | 2,77 | 2,98 |
| **CD19** | 42,6 | 7,28 | 15,13 | 9,28 | 2,82 |
| **IL21** | 33,0 | 6,05 | 12,05 | 5,64 | 2,77 |
| **COLEC12** | 12,7 | 1,81 | 4,81 | 2,12 | 2,63 |
| **MAP3K7IP1** | 25,9 | 4,48 | 10,63 | 1,98 | 2,44 |
| **BLK** | 7,9 | 2,18 | 3,28 | 1,56 | 2,40 |
| **CCR7** | 17,4 | 3,15 | 7,52 | 3,25 | 2,32 |
| **TCL1A** | 11,8 | 3,03 | 5,29 | 2,50 | 2,24 |
| **CD1C** | 24,4 | 5,83 | 11,01 | 4,41 | 2,22 |
| **CD80** | 45,7 | 9,90 | 21,17 | 13,71 | 2,16 |
| **CD79A** | 22,0 | 4,50 | 10,22 | 4,57 | 2,15 |
| **LCK** | 9,0 | 1,46 | 4,32 | 1,86 | 2,09 |
| **DOK3** | 9,2 | 2,48 | 4,55 | 0,80 | 2,01 |
| **BISULFITE PYROSEQUENCING** | | | | | |
| **GENES** | **%METH. RBL** | ***SD*** | **%METH. LCL** | ***SD*** | **FC** |
| **CCL3L1** | 22,89 | 4,17 | 0,12 | 0,20 | 188,11 |
| **FCER2** | 26,77 | 4,73 | 2,53 | 0,51 | 10,57 |
| **SLAMF7** | 44,81 | 8,11 | 3,78 | 1,05 | 11,87 |
| **BLNK** | 21,23 | 4,83 | 2,14 | 0,42 | 9,94 |
| **IL25** | 34,85 | 6,57 | 5,31 | 3,40 | 6,56 |
| **IRS2** | 6,22 | 2,14 | 1,39 | 0,31 | 4,49 |
| **TRAF1** | 21,15 | 3,80 | 1,99 | 0,31 | 10,64 |
| **TAP1** | 27,92 | 6,62 | 4,85 | 2,93 | 5,76 |
| **CD19** | 7,86 | 2,49 | 1,33 | 0,24 | 5,91 |
| **IL21** | 47,47 | 6,08 | 17,93 | 5,47 | 2,65 |
| **COLEC12** | 6,88 | 0,87 | 3,00 | 1,16 | 2,29 |
| **MAP3K7IP1** | 47,76 | 6,42 | 16,61 | 2,35 | 2,87 |
| **BLK** | 8,72 | 2,62 | 2,12 | 0,37 | 4,12 |
| **CCR7** | 17,64 | 3,12 | 4,27 | 1,95 | 4,14 |
| **TCL1A** | 17,32 | 4,36 | 2,98 | 1,21 | 5,81 |
| **CD1C** | 23,10 | 3,91 | 7,66 | 3,88 | 3,02 |
| **CD80** | 53,45 | 5,35 | 15,29 | 2,90 | 3,50 |
| **CD79A** | 34,11 | 3,76 | 14,01 | 6,72 | 2,43 |
| **LCK** | 22,35 | 2,36 | 9,85 | 6,56 | 2,27 |
| **DOK3** | 6,91 | 2,06 | 2,27 | 0,47 | 3,04 |
