# Supplementary figures and images for: The B cell transcription program mediates hypomethylation and overexpression of key genes in Epstein-Barr virus-associated proliferative conversion
Source: Genome Biol. 2013 Jan 15;14(1):R3. doi: 10.1186/gb-2013-14-1-r3 (PMC3663113; doi:10.1186/gb-2013-14-1-r3)

# Additional file 3

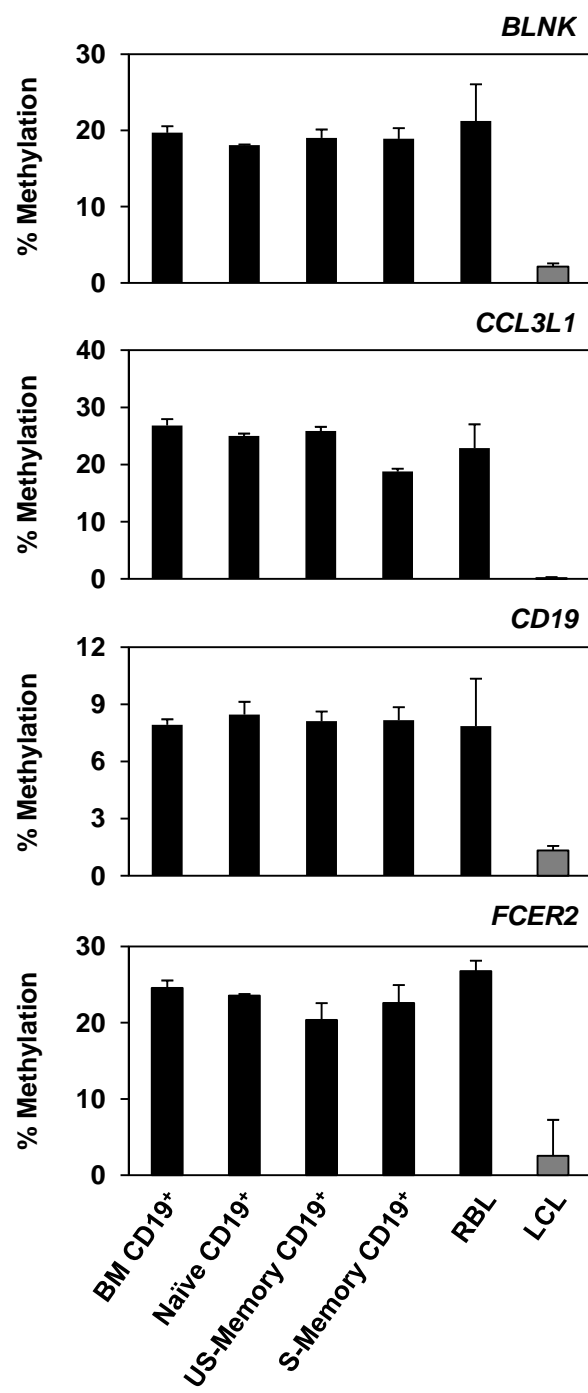

Supplement: Additional file 3 — A comparison of the DNA methylation levels of selected genes in different B cell types. Bisulfite pyrosequencing was performed for the genes selected from experiments with methylation arrays. The analysis includes bone marrow (BM) CD19+ cells, naïve B cells, unswitched (US) memory B cells and switched (S) memory cells. Also peripheral blood resting B cells (RBLs) and corresponding lymphoblastoid B cells (LCLs) are included. [file gb-2013-14-1-r3-S3.PDF]
