# Supplementary material for: The B cell transcription program mediates hypomethylation and overexpression of key genes in Epstein-Barr virus-associated proliferative conversion
Source: Genome Biol. 2013 Jan 15;14(1):R3. doi: 10.1186/gb-2013-14-1-r3 (PMC3663113; doi:10.1186/gb-2013-14-1-r3)
Supplement: Additional file 4 — Expression changes between those undergoing hypomethylation during the conversion between resting B cells and lymphoblastoid cells. [file gb-2013-14-1-r3-S4.DOCX]

**Additional file 4**

**Expression changes between those undergoing hypomethylation during the conversion between resting B cells and Lymphoblastoid cells**

| **RefSeq mRNA** | **Gene Name** | **B CELLS** | **LCL** | **Differential Expression_LCL/B_(Log2FC)** |
| --- | --- | --- | --- | --- |
| NM_005755 | EBI3 | 8.361579 | 11.8806079 | **3.5190289** |
| NM_000595 | LTA | 9.9914428 | 13.2446134 | **3.2531706** |
| NM_003037 | SLAMF1 | 9.3015445 | 12.4300744 | **3.1285299** |
| NM_144646 | IGJ | 10.6630748 | 13.0998334 | **2.4367586** |
| NM_005191 | CD80 | 7.2550475 | 9.3978496 | **2.1428021** |
| NM_021181 | SLAMF7 | 7.0929 | 9.0079051 | **1.9150051** |
| NM_003302 | TRIP6 | 8.4083063 | 10.1202375 | **1.7119312** |
| NM_020230 | PPAN | 7.7753293 | 9.3978573 | **1.622528** |
| NM_024508 | ZBED2 | 6.9995846 | 8.5368307 | **1.5372461** |
| NM_000593 | TAP1 | 12.3240457 | 13.8610525 | **1.5370068** |
| NM_014350 | TNFAIP8 | 9.5736415 | 11.0848396 | **1.5111981** |
| NM_016428 | ABI3 | 8.5261567 | 10.0101785 | **1.4840218** |
| NM_015946 | PELO | 9.7186697 | 11.1334539 | **1.4147842** |
| NM_001001437 | CCL3L3 | 10.7777109 | 12.0734895 | **1.2957786** |
| NM_005658 | TRAF1 | 7.2408395 | 8.5293985 | **1.288559** |
| NM_005018 | PDCD1 | 7.11799 | 8.3719593 | **1.2539693** |
| NM_001242 | CD27 | 9.7038254 | 10.9118643 | **1.2080389** |
| NM_005662 | VDAC3 | 11.8168899 | 12.9832593 | **1.1663694** |
| NM_023003 | TM6SF1 | 8.700683 | 9.7974065 | **1.0967235** |
| NM_006818 | MLLT11 | 8.6416641 | 9.7229634 | **1.0812993** |
| NM_004833 | AIM2 | 8.5478634 | 9.6275398 | **1.0796764** |
| NM_031917 | ANGPTL6 | 7.7481466 | 8.8171395 | **1.0689929** |
| NM_005683 | GPR55 | 7.1264973 | 8.1830253 | **1.056528** |
| NM_005290 | GPR15 | 7.0547916 | 8.0741712 | **1.0193796** |
| NM_030641 | APOL6 | 7.1144994 | 8.0574326 | **0.9429332** |
| NM_002201 | ISG20 | 12.9491047 | 13.8529576 | **0.9038529** |
| NM_001004419 | CLEC2D | 8.0498586 | 8.9124286 | **0.86257** |
| NM_014206 | C11orf10 | 12.9256825 | 13.7860901 | **0.8604076** |
| NM_013321 | SNX8 | 9.319911 | 10.164525 | **0.844614** |
| NM_021006 | CCL3L1 | 9.1810205 | 10.0247684 | **0.8437479** |
| NM_018843 | SLC25A40 | 7.9126 | 8.7266553 | **0.8140553** |
| NM_002245 | KCNK1 | 6.9683215 | 7.7384548 | **0.7701333** |
| NM_001838 | CCR7 | 11.0318769 | 11.7616788 | **0.7298019** |
| NM_052969 | RPL39L | 8.7735585 | 9.442486 | **0.6689275** |
| NM_000265 | NCF1 | 12.0732979 | 12.7276262 | **0.6543283** |
| NM_022745 | ATPAF1 | 9.3267579 | 9.956458 | **0.6297001** |
| NM_003290 | TPM4 | 8.447076 | 9.0500683 | **0.6029923** |
| NM_152866 | MS4A1 | 7.7155858 | 8.3140804 | **0.5984946** |
| NM_005114 | HS3ST1 | 7.0427225 | 7.575944 | **0.5332215** |
| NM_024819 | DCAKD | 9.5121512 | 10.0413891 | **0.5292379** |
| NM_015497 | TMEM87A | 11.0309814 | 11.55017 | **0.5191886** |
| NM_032797 | AIFM2 | 7.3816228 | 7.8797569 | **0.4981341** |
| NM_024872 | DOK3 | 7.9673326 | 8.461666 | **0.4943334** |
| NM_001733 | C1R | 7.0333089 | 7.5221181 | **0.4888092** |
| NM_003612 | SEMA7A | 7.8964416 | 8.3464553 | **0.4500137** |
| NM_002231 | CD82 | 9.7770887 | 10.2259283 | **0.4488396** |
| NM_001289 | CLIC2 | 7.4940556 | 7.929496 | **0.4354404** |
| NM_014238 | KSR1 | 7.3013077 | 7.7221951 | **0.4208874** |
| NM_003778 | B4GALT4 | 8.1650151 | 8.58495 | **0.4199349** |
| NM_207646 | LOC400696 | 6.907167 | 7.2862295 | **0.3790625** |
| NM_030781 | COLEC12 | 7.0815068 | 7.4383001 | **0.3567933** |
| NM_031280 | MRPS15 | 11.0127227 | 11.359987 | **0.3472643** |
| NM_005356 | LCK | 8.0683004 | 8.4092464 | **0.340946** |
| NM_080764 | ZNF280B | 7.1542815 | 7.47718 | **0.3228985** |
| NM_024943 | TMEM156 | 9.0928819 | 9.4003039 | **0.307422** |
| NM_022895 | C12orf43 | 8.6127391 | 8.9137032 | **0.3009641** |
| NM_000093 | COL5A1 | 7.0073636 | 7.3078245 | **0.3004609** |
| NM_022036 | GPRC5C | 7.1469192 | 7.4201077 | **0.2731885** |
| NM_016337 | EVL | 12.8389397 | 13.0900986 | **0.2511589** |
| NM_005084 | PLA2G7 | 9.0243537 | 9.2506019 | **0.2262482** |
| NM_177986 | DSG4 | 7.0886668 | 7.2809235 | **0.1922567** |
| NM_013314 | BLNK | 7.1143012 | 7.306251 | **0.1919498** |
| NM_007237 | SP140 | 9.6892357 | 9.8571137 | **0.167878** |
| NM_032740 | SFT2D3 | 7.5296076 | 7.6802318 | **0.1506242** |
| NM_022490 | POLR1E | 9.4464953 | 9.5948265 | **0.1483312** |
| NM_002264 | KPNA1 | 8.8513366 | 8.9990582 | **0.1477216** |
| NM_002298 | LCP1 | 14.0244426 | 14.1653381 | **0.1408955** |
| NM_152495 | CNIH3 | 7.0570756 | 7.188799 | **0.1317234** |
| NM_173847 | SPACA3 | 7.1514022 | 7.268476 | **0.1170738** |
| NM_181616 | KRTAP20-2 | 7.1243532 | 7.2292474 | **0.1048942** |
| NM_004597 | SNRPD2 | 10.6874439 | 10.7890669 | **0.101623** |
| NM_198083 | DHRS4L2 | 8.9122288 | 8.98009 | **0.0678612** |
| NM_002506 | NGF | 7.2157388 | 7.2566406 | **0.0409018** |
| NM_002988 | CCL18 | 7.2161696 | 7.2499725 | **0.0338029** |
| NM_001420 | ELAVL3 | 7.0617582 | 7.0944681 | **0.0327099** |
| NM_005559 | LAMA1 | 7.1620112 | 7.1895857 | **0.0275745** |
| NM_002845 | PTPRM | 7.2949795 | 7.3164616 | **0.0214821** |
| NM_018176 | LGI2 | 7.1926555 | 7.1853956 | **-0.0072599** |
| NM_144604 | ZC3H18 | 8.0379618 | 8.0293888 | **-0.008573** |
| NM_000681 | ADRA2A | 7.2658843 | 7.2424543 | **-0.02343** |
| NM_003728 | UNC5C | 7.2654913 | 7.2401594 | **-0.0253319** |
| NM_013434 | KCNIP3 | 7.3027624 | 7.2652581 | **-0.0375043** |
| NM_182617 | ACSM2B | 7.0752929 | 7.0374468 | **-0.0378461** |
| NM_138999 | NETO1 | 7.1705506 | 7.1274498 | **-0.0431008** |
| NM_153497 | TAB1 | 8.5717969 | 8.5280448 | **-0.0437521** |
| NM_003562 | SLC25A11 | 8.4017002 | 8.3573977 | **-0.0443025** |
| NM_001005463 | EBF3 | 7.0802674 | 7.0200855 | **-0.0601819** |
| NM_002348 | LY9 | 9.0255636 | 8.9595487 | **-0.0660149** |
| NM_018836 | AJAP1 | 7.2814544 | 7.2090507 | **-0.0724037** |
| NM_002664 | PLEK | 12.9644102 | 12.88791 | **-0.0765002** |
| NM_002410 | MGAT5 | 7.5339454 | 7.4572648 | **-0.0766806** |
| NM_019885 | CYP26B1 | 7.236905 | 7.1380913 | **-0.0988137** |
| NM_145657 | GSX1 | 7.1303837 | 7.0301804 | **-0.1002033** |
| NM_005987 | SPRR1A | 7.3654864 | 7.2507301 | **-0.1147563** |
| NM_033225 | CSMD1 | 7.36411 | 7.2389986 | **-0.1251114** |
| NM_015537 | NELF | 8.4546644 | 8.3260897 | **-0.1285747** |
| NM_014629 | ARHGEF10 | 7.3214536 | 7.1805581 | **-0.1408955** |
| NM_198082 | CCDC57 | 7.2285579 | 7.0838522 | **-0.1447057** |
| NM_014439 | IL37 | 8.3205202 | 8.1610922 | **-0.159428** |
| NM_006669 | LILRB1 | 10.2319596 | 10.0677938 | **-0.1641658** |
| NM_002002 | FCER2 | 11.1716663 | 10.9833388 | **-0.1883275** |
| NM_006258 | PRKG1 | 7.3748348 | 7.1645516 | **-0.2102832** |
| NM_012395 | CDK14 | 8.7201516 | 8.4830565 | **-0.2370951** |
| NM_005978 | S100A2 | 7.792796 | 7.5538529 | **-0.2389431** |
| NM_173803 | MPV17L | 7.4724232 | 7.2332966 | **-0.2391266** |
| NM_014587 | SOX8 | 7.4756184 | 7.2242305 | **-0.2513879** |
| NM_018948 | ERRFI1 | 8.0983696 | 7.825349 | **-0.2730206** |
| NM_020689 | SLC24A3 | 7.4052407 | 7.1240168 | **-0.2812239** |
| NM_021058 | HIST1H2BJ | 10.1041571 | 9.8181415 | **-0.2860156** |
| NM_002513 | NME3 | 10.897249 | 10.600442 | **-0.296807** |
| NM_021173 | POLD4 | 10.1476902 | 9.8340877 | **-0.3136025** |
| NM_181443 | BTBD3 | 7.6672246 | 7.3336139 | **-0.3336107** |
| NM_016205 | PDGFC | 7.4331173 | 7.0867396 | **-0.3463777** |
| NM_015431 | TRIM58 | 7.5231576 | 7.1623519 | **-0.3608057** |
| NM_001783 | CD79A | 12.6740692 | 12.3120288 | **-0.3620404** |
| NM_153265 | EML3 | 10.868667 | 10.4957541 | **-0.3729129** |
| NM_015364 | LY96 | 12.3529299 | 11.9249971 | **-0.4279328** |
| NM_001770 | CD19 | 12.2572695 | 11.8027973 | **-0.4544722** |
| NM_024910 | ZNF767 | 8.470725 | 7.9993678 | **-0.4713572** |
| NM_032831 | ORAI2 | 8.191573 | 7.7110509 | **-0.4805221** |
| NM_005320 | HIST1H1D | 7.5291347 | 7.0458345 | **-0.4833002** |
| NM_001715 | BLK | 11.0937337 | 10.596271 | **-0.4974627** |
| NM_005955 | MTF1 | 9.4440521 | 8.8959327 | **-0.5481194** |
| NM_006354 | TADA3 | 9.0802573 | 8.5050278 | **-0.5752295** |
| NM_001817 | CEACAM4 | 7.7185773 | 7.0888616 | **-0.6297157** |
| NM_001621 | AHR | 10.1039124 | 9.4537515 | **-0.6501609** |
| NM_006480 | RGS14 | 8.7010832 | 8.0351807 | **-0.6659025** |
| NM_001397 | ECE1 | 7.9643032 | 7.2916832 | **-0.67262** |
| NM_000250 | MPO | 7.7354272 | 7.0118387 | **-0.7235885** |
| NM_002612 | PDK4 | 7.7025053 | 6.9586864 | **-0.7438189** |
| NM_001725 | BPI | 8.0841175 | 7.3345391 | **-0.7495784** |
| NM_004949 | DSC2 | 7.8193279 | 7.0659212 | **-0.7534067** |
| NM_183376 | ARRDC4 | 8.2814058 | 7.333903 | **-0.9475028** |
| NM_002068 | GNA15 | 10.2910125 | 9.1692301 | **-1.1217824** |
| NM_173558 | FGD2 | 12.1054585 | 10.8484514 | **-1.2570071** |
| NM_012307 | EPB41L3 | 9.1316059 | 7.8604059 | **-1.2712** |
| NM_000634 | CXCR1 | 8.5159821 | 7.1581917 | **-1.3577904** |
| NM_013416 | NCF4 | 9.5309772 | 8.1685649 | **-1.3624123** |
| NM_001782 | CD72 | 9.8806107 | 8.3856939 | **-1.4949168** |
| NM_032310 | C9orf89 | 10.8653402 | 8.9445968 | **-1.9207434** |
| NM_170773 | RASSF2 | 11.7860975 | 9.8567473 | **-1.9293502** |
| NM_004951 | GPR183 | 12.5094472 | 10.0147594 | **-2.4946878** |
| NM_000099 | CST3 | 13.5770014 | 11.0284567 | **-2.5485447** |
| NM_001765 | CD1C | 10.0041464 | 7.3601415 | **-2.6440049** |
| NM_015714 | G0S2 | 11.4836496 | 7.9780711 | **-3.5055785** |
| NM_001945 | HBEGF | 11.5997406 | 8.0115404 | **-3.5882002** |
| NM_003749 | IRS2 | 11.6583416 | 7.7414119 | **-3.9169297** |
