# Supplementary material for: The B cell transcription program mediates hypomethylation and overexpression of key genes in Epstein-Barr virus-associated proliferative conversion
Source: Genome Biol. 2013 Jan 15;14(1):R3. doi: 10.1186/gb-2013-14-1-r3 (PMC3663113; doi:10.1186/gb-2013-14-1-r3)
Supplement: Additional file 5 — Primer sequences. [file gb-2013-14-1-r3-S5.DOC]

**Additional file 5. Primer sequences**

| **GENE** | **TYPE** | **FORWARD** | **REVERSE** |  |
| --- | --- | --- | --- | --- |
| Blue | Adapter AUMA | CCGAATTCGCAAAGCTCTGA |  |  |
| P-MCA | Adapter AUMA | ^P^-TCAGAGCTTTGCGAAT |  |  |
| ALU up 5 | AUMA | ATTCGCAAAGCTCTGAGGGTT |  |  |
|  |  |  |  |  |
| *QUEST* | 5hmC | CAAGGATCGCTCGCGGCTCTTA | AGGCAGACAAGGTATAGGGCG |  |
| *BLNK* | 5hmC | CTCCCTAGAGCAGGGGTGTT | CCTAGGGAGCAGCATGGTAA |  |
| *CCR7* | 5hmC | AGGGGCTTGAACCACTTCCTCCC | CATGACGCTCTCTGGGCGGT |  |
| *CD19* | 5hmC | TTACCCTCTCTGAGCCTCCA | GCAGAGGACTCCAAAAGCTG |  |
| *FCER2* | 5hmC | AATCTCATGGTGGGGTGTCT | ACGGAAGGCTCTAGGCAGA |  |
| *TRAF1* | 5hmC | TGCATTTGTTTAGCGCTTTG | CACCCCACTCAGGAAGTCAT |  |
|  |  |  |  |  |
| *HPR1* | RT-PCR | TGACACTGGCAAAACAATGCA | GGTCCTTTTCACCAGCAAGCT |  |
| *b-ACTIN* | RT-PCR | ATCGTCCACCGCAAATGCTTCTA | AGCCATGCCAATCTCATCTTGTT |  |
| *AID* | RT-PCR | AGAGGCGTGACAGTGCTACA | TGTAGCGGAGGAAGAGCAAT |  |
| *BLK* | RT-PCR | CTCTGTATGACTACACCGCT | AAGAACCACCTTTCCATTTCC |  |
| *BLNK* | RT-PCR | ACCAGAGGCTTACCATGCTG | CCTTCGAGGAACACTTGGAG |  |
| *CCR7 (EBI1)* | RT-PCR | GTGGTGGCTCTCCTTGTCAT | GCTTTAAAGTTCCGCACGTC |  |
| *CD1C* | RT-PCR | TGGGTGCCATCTCCAGGCTGT | GGCTTCTGGCCTCACTTGCCT |  |
| CD79A | RT-PCR | AGGGCTCCTGAGAGGTTTGT | GGCTGTGATGATTCGGTTCT |  |
| DOK3 | RT-PCR | GTCCCCATGGAGGAAAACTC | AAGTGGTAGGGCCAGCTGTA |  |
| EBI3 | RT-PCR | CCTGCAGTGGAAGGAAAGG | CTGTACGTGGCAATGAAGGA |  |
| FCER2 | RT-PCR | GGGAGGAGGTGACAAAGCTA | CCATGTCGTCACAGGCATAC |  |
| GPR183 (EBI2) | RT-PCR | GACCCGAACGAGTCACTGAT | ACGAGCCCAATGATGAAGAC |  |
| *LCK* | RT-PCR | TGAGAACTGCCATTATCCCA | ATAACCAGGTTGTCTTGCAG |  |
| *MAP3K7IP1* | RT-PCR | CAATGTCGGTACAAACCGTG | GCTTGATCTTTCCAGCATCC |  |
| TRAF1 | RT-PCR | TCACCCAGACACTCCAAACA | CAGGGATGGAGCAGGAATTA |  |
|  |  |  |  |  |
| *CCL3L1* | BS sequencing | GTAGTTGGGATTATAGGTATGTGTT | CTCTCAACTCTCAACTCATAACTAA |  |
| *FCER2* | BS sequencing | ATTTGATTTGGGAGTTTATTTG | AACAAATTCCCACAAAACTAAA |  |
| *EBV-Wp1* | BS sequencing | AGTTTTAGGGAGGGGGATTATT | TTAAAATCCACTTACCTCTAACCC |  |
| *EBV-Wp2* | BS sequencing | AATTTTTGGTAGTGATTTGGAT | AAAATAAAAAACCCCCTCTTAC |  |
|  |  |  |  |  |
| **GENE** | **TYPE** | **FORWARD** | **REVERSE** | **SEQUENCING** |
| *BLK* | Pyrosequencing | AAAGTAAAATTAGTGAGGTTGAAAGAA | ACAAACCTCCATACTAACTCTACAAAA | AACTCTACAAAATAATTTACCC |
| *BLNK* | Pyrosequencing | TTTTTAGGAGGGTTAGGAATTGT | AACAAAACATTCCAAAATTTCAATA | AGAATGGATAAGTTTAATAAAA |
| *CCL3L1* | Pyrosequencing | TTTTGGTTAGGTTGGTTTTAAATT | TATATCAACCCAAAACTATTCTTAATT | GTTGGGATTATAGGTGTG |
| *CCR7* | Pyrosequencing | TTTATGGTTTGGTATGTGATATTTATG | CCCTTCAATACCTTCAAAAATAAAA | AATCATTAAATCATTTACATTC |
| *CD19* | Pyrosequencing | GTTTTTTGTTTTATAATTTGGTGTGAG | AATCCCCTTAAAACACTACAACACA | GGAGGTTTTTTTTATTTATG |
| *CD1C* | Pyrosequencing | TTTTTTTTTTAGGTGGTGATAATGTAG | TTCCTTACACAAAACCCATACTCTA | TTTTTAGGTGGTGATAATG |
| *CD79A* | Pyrosequencing | GGGTTGGGATTATAGGTATAAGTTATT | AAACCAAAATATCTCCCTATCTCAT | TTTTAGTAAAGAGTTGATTATG |
| *CD80* | Pyrosequencing | TGGGAGTGATGGGTATGATTAAAGT | CCACCTCTAAATCCCACCATCTT | AATCCCACCATCTTCA |
| *COLEC12* | Pyrosequencing | TTAGGTAGTTTGGGGTGTAGAA | AACTCTCTCTTTTTCCCRTTAC | GGTAGTTTGGGGTGTAGA |
| *DOCK3* | Pyrosequencing | TGGGGTTATTTAGGGTTGTG | AAAACCCCCTCACCACTATACT | TTTTATGTGGATTGGATG |
| *FCER2* | Pyrosequencing | GGGTGGTAGGATTTTAATTTTAGGT | TAAACAACAAATTCCCACAAAACT | GGTAGGATTTTAATTTTAGGT |
| *IL21* | Pyrosequencing | GGGTTTTATTTTTTTTTGAAGGATGA | ACAAATAACAATCCTCTCCATATTACC | TTGAAGGATGAATAAATAGG |
| *IL25* | Pyrosequencing | GAAGGTGGATGATTAGAGGGATAT | TTATAACCTCCCTTCAAAAAACC | AGAGGGGAATGGTGA |
| *IRS2* | Pyrosequencing | GTTYGGGGAGAAAGGGG | CCRCACAATAAATAACACATC | TTGTTTTGTTTTTTTTAGTTT |
| *LCK* | Pyrosequencing | GGTGTTGTTTTGGTTTTTGATTAT | CCATCCCTCTTACAAACAAAATACT | TTGTTTTGGTTTTTGATTA |
| *MAP3K7IP1* | Pyrosequencing | TTAGTTTTTTGAAGGTTTGTTTTGT | ACCAAACTAATCTTAAACTCCTAAA | TAACCCTACTTAACTCTATAAT |
| *SLAMF7* | Pyrosequencing | GTTTTATTTTGGGTTTTTGGTTTAA | CTTCATCCCTAAAACAATCAACA | AAATAAAAACACAAAACCTC |
| *TAP1* | Pyrosequencing | TGAGTTGGAAGGAGTTTTAAAGAT | AAACCTAAATTTTAATCCCAAATCTAC | TGGAAGGAGTTTTAAAGAT |
| *TCL1A* | Pyrosequencing | TTTGTGGGTTTGGGAGAA | AACTCCCAAAAACTACCCCTACT | GGGTTTGGGAGAAGTT |
| *TRAF1* | Pyrosequencing | TTGTTTAGAGGTGTTAGGGAATATAGA | AACACAATAAAAATTCCCCTAATTAAA | AATATAGATTAGGGATTAGTTG |
