# Supplementary material for: The B cell transcription program mediates hypomethylation and overexpression of key genes in Epstein-Barr virus-associated proliferative conversion
Source: Genome Biol. 2013 Jan 15;14(1):R3. doi: 10.1186/gb-2013-14-1-r3 (PMC3663113; doi:10.1186/gb-2013-14-1-r3)
Supplement: Additional file 6 — Individual raw data corresponding to bisulfite pyrosequencing presented in Figure 2. [file gb-2013-14-1-r3-S6.DOCX]

**Additional file 6. Individual raw data corresponding to bisulfite pyrosequencing presented in Figure 2.** Individual raw data corresponding to bisulfite pyrosequencing of individual RBL and LCL samples (top) (Figure 2A) and time course methylation data in 3 samples (P1, P2, P3) over time (0, 1, 3, 24 h; 7 days, 2 weeks) (bottom) (Figure 2C). Data are presented as supplied by PyroMark Q24 Software 2.0 (Qiagen), which automatically generates methylation percentages in a datasheet format.

|  |  | **RBL samples** | | | | | | **LCL samples** | | | | | | **RBL** | | **LCL** | |
| --- | --- | --- | --- | --- | --- | --- | --- | --- | --- | --- | --- | --- | --- | --- | --- | --- | --- |
| **Gene Name** | **Map info** | **H2.1** | **H3.1** | **H4.1** | **H5.1** | **H6.1** | **H7.1** | **H2.2** | **H3.2** | **H4.2** | **H5.2** | **H6.2** | **H7.2** | **Average** | **Desvprom** | **Average** | **Desvprom** |
| ***CCL3L1*** | 31650167 | 27.24 | 27.79 | 12.47 | 22.31 | 26.14 | 21.37 | 0.00 | 0.00 | 0.00 | 0.73 | 0.00 | 0.00 | 22.89 | 4.17 | 0.12 | 0.20 |
| ***FCER2*** | 7673348 | 29.95 | 35.22 | 19.51 | 24.63 | 29.33 | 22.00 | 1.23 | 2.57 | 3.11 | 3.14 | 2.30 | 2.85 | 26.77 | 4.73 | 2.53 | 0.51 |
| ***SLAMF7*** | 158975863 | 56.99 | 54.65 | 24.38 | 47.12 | 44.76 | 40.98 | 2.43 | 5.30 | 2.34 | 3.65 | 5.40 | 3.53 | 44.81 | 8.11 | 3.78 | 1.05 |
| ***BLNK*** | 98021115 | 22.68 | 30.14 | 13.73 | 20.38 | 25.36 | 15.11 | 2.53 | 2.74 | 1.75 | 2.08 | 1.31 | 2.41 | 21.23 | 4.83 | 2.14 | 0.42 |
| ***IL25*** | 22911594 | 46.15 | 42.88 | 18.09 | 35.23 | 32.49 | 34.25 | 1.43 | 9.16 | 7.18 | 1.94 | 9.78 | 2.36 | 34.85 | 6.57 | 5.31 | 3.40 |
| ***IRS2*** | 109236938 | 2.84 | 5.51 | 4.23 | 10.35 | 8.52 | 5.87 | 0.93 | 1.86 | 1.58 | 1.28 | 1.02 | 1.65 | 6.22 | 2.14 | 1.39 | 0.31 |
| ***TRAF1*** | 122728536 | 28.29 | 23.53 | 11.12 | 22.15 | 19.78 | 22.05 | 1.78 | 1.62 | 2.37 | 1.90 | 1.71 | 2.55 | 21.15 | 3.80 | 1.99 | 0.31 |
| ***TAP1*** | 32931094 | 37.63 | 39.12 | 18.45 | 25.53 | 28.47 | 25.11 | 3.54 | 5.51 | 2.26 | 1.84 | 14.59 | 1.8 | 29.05 | 6.22 | 4.92 | 3.42 |
| ***CD19*** | 28851178 | 6.10 | 12.31 | 7.31 | 6.83 | 10.87 | 3.73 | 1.33 | 1.80 | 1.10 | 1.05 | 1.12 | 1.58 | 7.86 | 2.49 | 1.33 | 0.24 |
| ***IL21*** | 123761648 | 56.58 | 54.62 | 31.47 | 45.24 | 49.46 | 47.45 | 19.16 | 28.19 | 13.35 | 14.34 | 22.82 | 9.69 | 47.47 | 6.08 | 17.93 | 5.47 |
| ***COLEC12*** | 490979 | 5.80 | 5.89 | 6.76 | 8.47 | 7.90 | 6.44 | 1.64 | 2.51 | 2.04 | 5.53 | 3.96 | 2.32 | 6.88 | 0.87 | 3.00 | 1.16 |
| ***MAP3K7IP1*** | 38125134 | 55.71 | 53.10 | 28.92 | 52.31 | 47.34 | 49.16 | 12.20 | 16.89 | 16.36 | 20.31 | 19.70 | 14.22 | 47.76 | 6.42 | 16.61 | 2.35 |
| ***BLK*** | 11388262 | 7.50 | 13.87 | 8.65 | 6.71 | 11.42 | 4.16 | 1.94 | 2.42 | 1.62 | 2.03 | 1.76 | 2.92 | 8.72 | 2.62 | 2.12 | 0.37 |
| ***CCR7*** | 35975468 | 23.04 | 21.59 | 10.22 | 17.41 | 17.07 | 16.51 | 7.18 | 7.20 | 1.45 | 3.32 | 2.78 | 3.66 | 17.64 | 3.12 | 4.27 | 1.95 |
| ***TCL1A*** | 95250072 | 14.09 | 26.22 | 20.31 | 18.49 | 15.74 | 9.05 | 2.32 | 5.01 | 1.43 | 2.40 | 2.15 | 4.57 | 17.32 | 4.36 | 2.98 | 1.21 |
| ***CD1C*** | 156526550 | 27.40 | 28.84 | 17.01 | 24.79 | 22.14 | 18.42 | 3.37 | 12.69 | 3.81 | 6.43 | 14.26 | 5.41 | 23.10 | 3.91 | 7.66 | 3.88 |
| ***CD80*** | 120760511 | 41.47 | 57.13 | 60.12 | 50.39 | 59.16 | 52.43 | 11.79 | 18.11 | 11.41 | 13.97 | 17.23 | 19.23 | 53.45 | 5.35 | 15.29 | 2.90 |
| ***CD79A*** | 47072565 | 38.92 | 40.58 | 26.90 | 33.61 | 33.83 | 30.80 | 2.76 | 16.22 | 31.97 | 9.98 | 12.86 | 10.27 | 34.11 | 3.76 | 14.01 | 6.72 |
| ***LCK*** | 32489589 | 25.12 | 25.48 | 17.11 | 23.22 | 20.51 | 22.64 | 1.56 | 12.51 | 1.26 | 8.56 | 26.86 | 8.35 | 22.35 | 2.36 | 9.85 | 6.56 |
| ***DOK3*** | 176869969 | 5.07 | 11.09 | 5.90 | 6.70 | 8.91 | 3.81 | 2.36 | 2.92 | 1.20 | 2.35 | 2.86 | 1.95 | 6.91 | 2.06 | 0.64 | 0.47 |

|  |  | Sample P1 | | | | | | Sample P1 | | | | | | Sample P1 | | | | | |
| --- | --- | --- | --- | --- | --- | --- | --- | --- | --- | --- | --- | --- | --- | --- | --- | --- | --- | --- | --- |
|  | **Map info** | **P1 0 h** | **P1 1 h** | **P1 3h** | **P1 24h** | **P1 7 d** | **P1 2wk** | **P2 0 h** | **P2 1 h** | **P2 3 h** | **P2 24h** | **P2 7 d** | **P2 2wk** | **P3 0 h** | **P3 1 h** | **P3 3 h** | **P3 24h** | **P3 7 d** | **P3 2wk** |
| ***BLNK*** | 98021115 | 15.05 | 16.93 | 15.48 | 14.33 | 15.33 | 4.44 | 23.82 | 26.21 | 31.63 | 25.62 | 15.17 | 8.67 | 21.5 | 12.78 | 14.59 | 10.99 | 14.63 | 9.69 |
| ***CCL3L1*** | 31650167 | 39.17 | 38.33 | 37.12 | 38.75 | 19.37 | 3.23 | 46.84 | 44.59 | 43.31 | 44.54 | 11.3 | 5.68 | 27.46 | 25.72 | 27.18 | 24.92 | 19.26 | 8.81 |
| ***CD19*** | 28851178 | 3.8 | 3.9 | 3.55 | 3.89 | 2.58 | 0.9 | 2.995 | 2.93 | 2.675 | 2.8 | 2.455 | 1.17 | 2.19 | 1.96 | 1.8 | 1.71 | 2.33 | 1.44 |
| ***FCER2*** | 7672974 | 42.5 | 35.93 | 39.35 | 40.73 | 19.28 | 15.32 | 48.3 | 46 | 45.01 | 39.38 | 11.3 | 8.41 | 71.73 | 59.94 | 50.5 | 52.19 | 10.53 | 12.7 |

Mapinfo: Genome build 36, Source version 36.1
